# Evaluating clinical heterogeneity and predicting mortality in severely burned patients through unsupervised clustering and latent class analysis

**DOI:** 10.1038/s41598-023-40927-7

**Published:** 2023-08-21

**Authors:** Sungmin Kim, Jaechul Yoon, Dohern Kym, Jun Hur, Myongjin Kim, Jongsoo Park, Yong Suk Cho, Wook Chun, Dogeon Yoon

**Affiliations:** 1 Department of Surgery and Critical Care, Burn Center, Hangang Sacred Heart Hospital, Hallym University Medical Center, College of Medicine, Hallym University, 12, Beodeunaru-ro 7-gil, Youngdeungpo-gu, Seoul, 07247 Korea; 2 Burn Institutes, Hangang Sacred Heart Hospital, Hallym University Medical Center, 12, Beodeunaru-ro 7-gil, Youngdeungpo-gu, Seoul, 07247 Korea

**Keywords:** Biomarkers, Risk factors

## Abstract

Burn injuries often result in a high level of clinical heterogeneity and poor prognosis in patients with severe burns. Clustering algorithms, which are unsupervised methods that can identify groups with similar trajectories in patients with heterogeneous diseases, can provide insights into the mechanisms of the disease pathogenesis. This study aimed to analyze routinely collected biomarkers to understand their mortality prediction power, identify the clinical meanings or subtypes, and inform treatment decisions to improve the outcomes of patients with burns. This retrospective cohort study included patients aged ≥ 18 years who were admitted between January 2010 and December 2021. The patients were divided into four subgroups based on the time period of their admission: week 1, 2, 3, and 4. The study revealed that 22 biomarkers were evaluated, and the red blood cell distribution width, bicarbonate level, pH, platelets, and lymphocytes were significantly associated with the mortality risk. Latent class analysis further demonstrated that the pH, platelets, lymphocytes, lactate, and albumin demonstrated the lowest levels in the cluster with the highest risk of mortality, with the lowest levels of pH and lactate being particularly noteworthy in week 1 of the study. During the week 2, the pH and lymphocyte levels were demonstrated to be significant predictors of the mortality risk, whereas the lymphocyte and platelet counts were meaningful predictors in week 3. During week 4, pH, platelet count, and albumin level were important predictors of mortality risk. Analysis of routinely collected biomarkers using clustering algorithms and latent class analysis can provide valuable insights into the heterogeneity of burn injuries and improve the ability to predict disease progression and mortality. Our findings suggest that lactate levels are a better indicator of cellular hypoxia in the early stages of burn shock, whereas platelet and lymphocyte levels are more indicative of infections such as sepsis. Albumin levels are considered a better indicator of reduced nutritional loss with decrease in unhealed burn wounds; however, the pH levels reflect the overall condition of the patient throughout the study period. These findings can be used to inform treatment decisions and improve the outcomes of burn patients.

## Introduction

Burn injury is a type of critical trauma, which is associated with high morbidity and mortality^1^. These injuries often exhibit a high degree of clinical heterogeneity, and patients with severe burns are at risk of complications, such as sepsis, organ failure, and hypermetabolism, which can worsen their prognosis. As patients with burns are often in a critical condition, identifying the predictors of mortality is a key area of research that can help physicians better understand the disease process and make more informed treatment decisions.

In recent years, large amounts of data, or “big data,” have become available to physicians in the form of longitudinal datasets. The analysis of these datasets can provide valuable insights into the disease progression and mortality, and can be used to make more accurate predictions about the course of the disease^2,3^. One approach to analyze these data is through the use of clustering algorithms such as longitudinal k-means clustering, which can be used to classify clinical data into groups based on shared characteristics^4^. These algorithms are particularly useful for identifying groups with similar trajectories in heterogeneous medical conditions, as they can provide insight into the mechanisms underlying disease heterogeneity. Latent class analysis (LCA) is another statistical method used to reduce the heterogeneity in burn injuries. LCA is used to identify the subtypes of related cases or latent classes from multivariate categorical data. This is similar to the principal component analysis or factor analysis, which are used for reducing heterogeneity in quantitative data^5^.

This study mainly aimed to analyze routinely collected biomarkers to understand their mortality prediction power, identify clinical implications or subtypes, and assess disease trajectories in patients with burns. Our hypothesis centers on the potential of these biomarkers to predict the mortality risk and reveal specific patterns using clustering algorithms. By employing statistical techniques, such as LCA, we sought to deepen our understanding of burn injuries and enhance our ability to predict disease progression and mortality. This study aimed to inform treatment decisions and improve outcomes in patients with burns, thereby contributing to more personalized and effective care strategies.

## Materials and methods

### Study site and patients

This retrospective cohort study included patients aged 18 years or older who were admitted to the burn intensive care unit (BICU) of Hangang Sacred Heart Hospital, Hallym University Medical Center, within 24 h of having a burn injury between January 2010 and December 2021. The patients were divided into four subgroups based on the time period of their admission: week 1, 2, 3, and 4. The criteria for admission to the BICU were as follows: (1) partial-thickness burns affecting more than 20% of the total body surface area (TBSA) in adults or more than 10% of the TBSA in pediatric or older patients, (2) inhalation injury, (3) electrical burns, (4) pre-existing medical conditions that could increase the risk of complications or mortality, and (5) concomitant trauma that could increase the risk of morbidity or mortality. All the patients underwent chest radiography, and routine serum laboratory tests were performed at least every 3–4 days during their stay in the BICU.

### Data collection and missing values

In this study, clinical longitudinal data were retrieved from a clinical database warehouse at the Hangang Sacred Heart Hospital. These data were collected prospectively from the time of admission to either death (non-survival group) or discharge from the BICU (survival group). When biomarkers were measured multiple times daily, the worst values were recorded. We recorded demographic characteristics, such as age, sex, TBSA (calculated using a modified Lund and Browder chart), type of burn, length of stay in the BICU, and presence of inhalation injury (defined as a history of smoke exposure in a closed space and prolonged extrication or physical findings such as singed facial hair, carbonaceous deposits in the oropharynx or sputum, facial burns, and voice changes). We also collected routine laboratory test results, including complete blood counts, and electrolyte, routine chemistry, C-reactive protein, lactate dehydrogenase, and lactate levels. The primary outcome of this study was in-hospital 60-day mortality. The severity of injury was reported using the Abbreviated Burn Severity Index and rBaux index at admission, and the Hangang score^6^. Acute Physiology and Chronic Health Evaluation Score (APACHE) IV, and Sequential Organ Failure Assessment (SOFA) scores were calculated daily based on routine laboratory test results. The daily prevalence of sepsis and acute kidney injury (AKI) was assessed using the sepsis-3 criteria^7^ and AKIN criteria^8^. Missing values for these longitudinal variables were imputed using the Copy Mean method, which is a commonly used method for imputing missing data in longitudinal studies^9^. This method involves interpolating the values immediately surrounding the missing data using a straight line, and imputing the missing values using the last observation carried forward or the following observation carried backwards. The missing longitudinal data are shown in an additional file (Fig. S1). Biomarker outliers were addressed through a winsorization process in which the upper outliers were replaced by the 95th percentile, and the lower outliers were replaced by the 5th percentile. The study was approved by the Institutional Review Board of Hangang Sacred Heart Hospital, and the need for informed consent was waived because the study was retrospective and did not involve any interventions.

### Burn treatment protocol

The treatment of patients with burns was conducted in accordance with the established guidelines of the Burn Center at Hangang Sacred Heart Hospital. Fluid resuscitation was conducted in patients in accordance with either the Parkland formula (for burns affecting less than 40% of the TBSA) or Warden formula (for burns affecting 40% of the TBSA). In the absence of specific complications such as ileus or prolonged burn shock, early enteral nutrition was initiated within 48 h post-burn. Subsequently, nutritional support was tailored through weekly consultations with a nutritional support team. Daily pain-controlled wound dressings were applied, and initial burn wound excisions with allograft applications were typically completed within 5 days following the injury, followed by autografting procedures. Antibiotic therapy was guided by the culture results from the sputum, blood, urine, and wound samples. In cases where patients were identified as having sepsis, immediate measures were taken to conduct cultures, and broad-spectrum antibiotics were administered as an initial response. Once the culture results were obtained, antibiotic treatment was adjusted accordingly to target the identified pathogens. Continuous renal replacement therapy was considered in cases of renal dysfunction, and decisions were made in collaboration with a nephrologist. Inhalation injuries were diagnosed using a comprehensive approach that included patient history, physical examination, and fiberoptic bronchoscopy.

### Statistical analysis

Baseline demographic characteristics were reported as follows: continuous variables that were distributed normally are presented as means ± standard deviations, while those that were distributed non-normally are presented as medians (25th–75th interquartile range). The paired *t*-test or Wilcoxon signed-rank test was used to determine the differences between the two groups based on the normality of the data. Categorical variables are presented as percentages and were analyzed using the Chi-square or Fisher's exact test. Longitudinal biomarkers were clustered into four groups using the k-means method and the R-project program of the kmlShape package, which is an efficient method for clustering longitudinal data based on their shapes. The optimal number of clusters (three) was chosen based on clinical relevance^10^. Subsequently, each cluster was assigned a letter from A to C based on its mortality rate. We conducted a multiple logistic regression analysis using the classified biomarkers from clustering to evaluate the discrimination performance of mortality prediction for each group. Following the evaluation of the discrimination performance of mortality prediction for each group using multiple logistic regression, LCA was performed to identify patterns of burn injury using the significant variables identified through this analysis. The number of latent classes was chosen based on the Akaike and Bayesian information criteria (AIC and BIC). Univariate associations of each class with mortality and covariates were evaluated using the chi-square test. A *p*-value of < 0.05 (two-sided) was considered statistically significant. All the analyses were conducted using R-project version 4.2.2.

### Ethics approval and consent to participate

The study was conducted in accordance with the Declaration of Helsinki, and approval by the Institutional Review Board of Hangang Sacred Heart Hospital (HG2022-013). The requirement for informed consent was waived owing to the retrospective nature of the study.

## Results

### Study population

**Figure 1:**
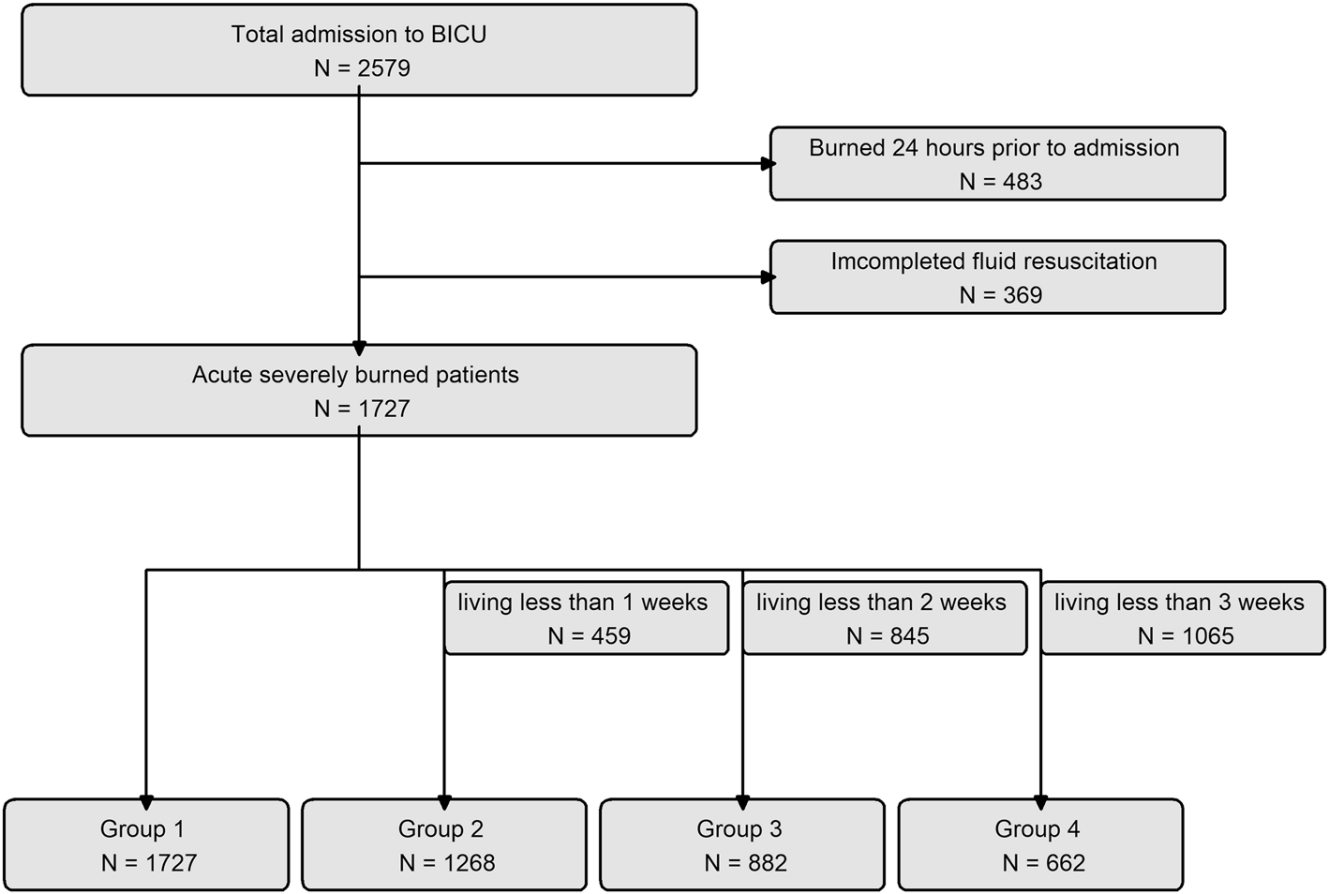
Flowchart illustrating the enrollment process for the study participants.

The present study analyzed the clinical characteristics and outcomes of 2579 patients admitted to the BICU between January 2010 and December 2021. A total of 483 patients were excluded because their admission occurred more than 24 h after the injury. Additionally, 369 patients were excluded because fluid resuscitation was not completed for them. Consequently, 1727 eligible patients meeting the criteria for this study were included in the analysis. The patients were systematically divided into four distinct groups based on their follow-up period in the ICU. These groups were categorized as follows: week 1, encompassing admission to 1 week; week 2, including those surviving for more than 1 week up to 2 weeks; week 3, for patients surviving longer than 2 weeks up to 3 weeks; and week 4, for those surviving longer than 3 weeks up to 4 weeks. The enrollment data for these patients are illustrated in Fig. 1, and the follow-up data corresponding to each group are detailed in Fig. S2.

**Figure 2:**
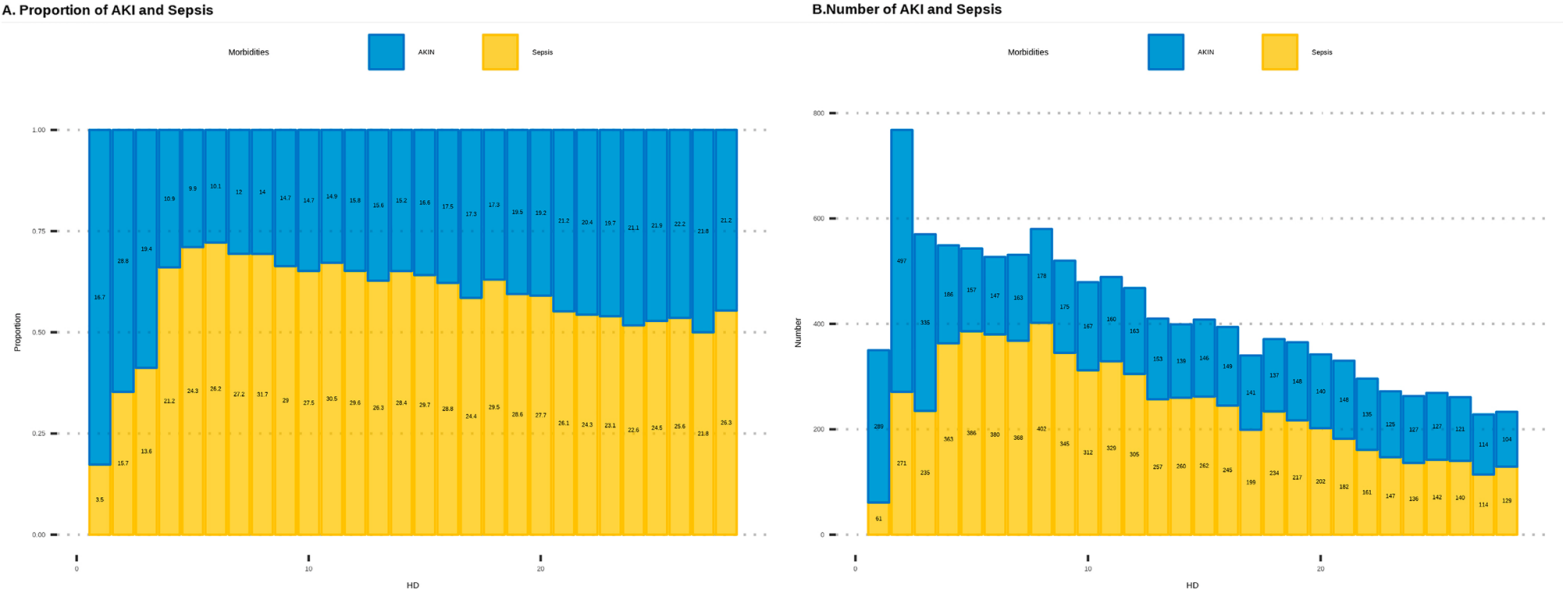
Prevalence of acute kidney injury (AKI) and sepsis during the study period.

The results of the study demonstrated that 22.5% of the patients (389 patients) died in week 1. The overall median age of the patients was 50 years (interquartile range [IQR] 40–60 years), and the majority were male (81.2%). The median TBSA was 30%, and inhalation injury was present in 44.2% of patients. The median length of stay in the BICU was 15 days (IQR 7–30 days), and the median APACHE IV, Abbreviated Burn Severity Index, rBaux, Hangang, and SOFA scores were 27, 8, 91, 132, and 3, respectively. Comorbidities included hypertension (16.5%), diabetes mellitus (7.5%), hyperlipidemia (2.6%), and cardiovascular disease (2.3%). The mortality rate was lower in week 4 (16.9%) than that in the other periods, and the length of ICU stay increased over the study period. The use of ventilators was highest in week 4 (70.1%), and the use of continuous renal replacement therapy (CRRT) was highest in week 1 (25.8%). The severity scores, except for the SOFA score, showed statistically significant differences between the groups (Table 1). The detailed results for each group are presented in Table S1. The mean prevalences of sepsis and AKI during the study period were 24.9% and 17.5%, respectively. The prevalence of AKI was high in the early stages of week 1, with the incidence of sepsis peaking thereafter and gradually declining over time (Fig. 2).

**Table 1:** Characteristics of enrolled patients in each of the four study groups.

Group	Variables	Week 1, N = 1727 (38.0%)	Week 2, N = 1268 (27.9%)	Week 3, N = 882 (19.4%)	Week 4, N = 662 (14.6%)	p-value
Demographics	Mortality	389 (22.5%)	304 (24.0%)	188 (21.3%)	112 (16.9%)	0.003
	Patient age					0.193
	Median [IQR]	50 [40, 60]	51 [41, 61]	51 [41, 61]	52 [42, 62]	
	Sex					0.362
	Male	1,402 (81.2%)	1,013 (79.9%)	696 (78.9%)	519 (78.4%)	
	Female	325 (18.8%)	255 (20.1%)	186 (21.1%)	143 (21.6%)	
	TBSA					< 0.001
	Median [IQR]	30 [19, 49]	34 [22, 52]	38 [24, 54]	38 [24, 54]	
	Type					0.085
	FB	1,274 (73.8%)	967 (76.3%)	683 (77.4%)	511 (77.2%)	
	SB	144 (8.3%)	110 (8.7%)	78 (8.8%)	60 (9.1%)	
	EB	234 (13.5%)	132 (10.4%)	78 (8.8%)	59 (8.9%)	
	ChB	23 (1.3%)	18 (1.4%)	13 (1.5%)	8 (1.2%)	
	CoB	52 (3.0%)	41 (3.2%)	30 (3.4%)	24 (3.6%)	
	Inhalation	764 (44.2%)	575 (45.3%)	397 (45.0%)	309 (46.7%)	0.758
	LOICU					< 0.001
	Median [IQR]	15 [7, 30]	23 [13, 36]	30 [22, 43]	36 [27, 49]	
	Apply of ventilator	1178 (68.2%)	773 (61.0%)	590 (66.9%)	464 (70.1%)	< 0.001
	Apply of CRRT	446 (25.8%)	90 (7.1%)	51 (5.8%)	44 (6.6%)	< 0.001
Severity scores	ABSI					< 0.001
	Median [IQR]	8 [6, 10]	9 [7, 10]	9 [7, 10]	9 [8, 10]	
	rBaux					< 0.001
	Median [IQR]	91 [72, 111]	96 [79, 115]	98 [82, 115]	99 [84, 115]	
	Hangang					< 0.001
	Median [IQR]	132 [121, 147]	123 [112, 136]	123 [112, 135]	124 [114, 136]	
	APACHE IV					< 0.001
	Median [IQR]	37 [23, 57]	37 [26, 52]	37 [28, 54]	40 [29, 56]	
	SOFA					0.304
	Median [IQR]	3 [2, 5]	3 [2, 5]	3 [2, 5]	3 [1, 5]	
Comorbidities	Hypertension	285 (16.5%)	222 (17.5%)	156 (17.7%)	123 (18.6%)	0.647
	Diabetes mellitus	130 (7.5%)	97 (7.6%)	63 (7.1%)	51 (7.7%)	0.970
	Tuberculosis	26 (1.5%)	22 (1.7%)	16 (1.8%)	14 (2.1%)	0.770
	Hepatobiliary	34 (2.0%)	23 (1.8%)	13 (1.5%)	11 (1.7%)	0.830
	Cardiovascular	39 (2.3%)	30 (2.4%)	22 (2.5%)	14 (2.1%)	0.963
	CVA	23 (1.3%)	22 (1.7%)	19 (2.2%)	14 (2.1%)	0.371
	Cancer	41 (2.4%)	28 (2.2%)	21 (2.4%)	15 (2.3%)	0.990
	Hyperlipidemia	45 (2.6%)	35 (2.8%)	23 (2.6%)	16 (2.4%)	0.977
	Other	432 (25.0%)	329 (25.9%)	234 (26.5%)	183 (27.6%)	0.587

### Prediction performance

**Figure 3:**
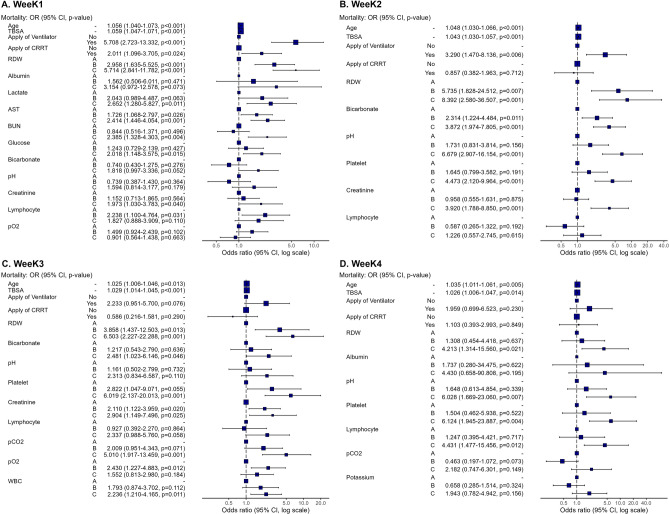
Multiple logistic analysis of mortality by time period (**A** Week 1, **B** Week 2, **C** Week 3, **D** Week 4).

In the present study, we evaluated 22 biomarkers that were checked at least every 4 days. These biomarkers included white blood cell count, hematocrit, platelets, red cell distribution width (RDW), neutrophils, lymphocytes, blood urea nitrogen, creatinine, aspartate aminotransferase, alanine aminotransferase, total bilirubin, albumin, glucose, creatine kinase, lactate dehydrogenase, pH, partial pressure of carbon dioxide, partial pressure of oxygen, bicarbonate, lactate, sodium, and potassium levels. Of these biomarkers, five (RDW, bicarbonate, pH, platelets, and lymphocytes) showed statistical significance for at least 3 weeks (Table S2). The odds ratios (ORs) for RDW were significant, except in cluster B at week 4. The overall *p*-value for bicarbonate was significant at week 1; however, the categorical *p*-values were not significant (Fig. 3 and Table S2). The OR plots for all the biomarkers are presented in Fig. 3. Considering CRRT and ventilation, only ventilation was significant at weeks 1 and 2. CRRT was significant at week 1 (Fig. 3).

### LCA

**Figure 4:**
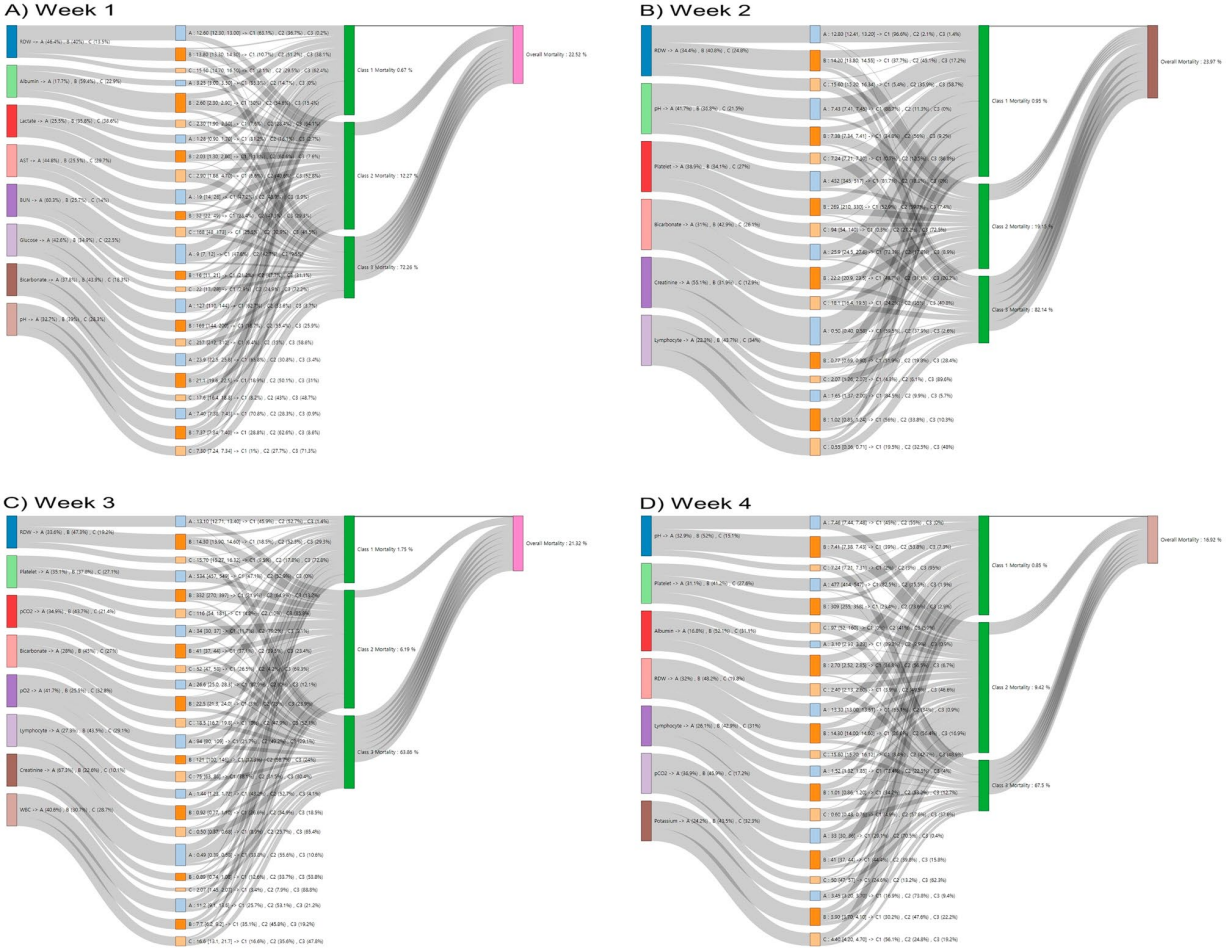
Sankey Diagram to explore the predictors, clusters, latent class, and mortality.

The highest utilization of CRRT was observed in Class 1, which was designated as a low-risk group during week 1, and in Class 3, which was characterized as a high-risk group during weeks 2–4. The highest usage of ventilators was recorded in Class 3 throughout the entire study period (Tables S3–S6). During week 1, class 3 was identified as a high-risk group for mortality (72.3%), which had a large proportion of cluster C individuals with high lactate levels (85.6%) and pH (84.7%) (Table S3). In week 2, class 3 individuals had a mortality rate of 82.1%, and a high proportion of cluster C individuals had low pH (86.4%), and platelets (88.6%) and lymphocyte (73.9%) levels (Table S4). In week 3, class 3 had a mortality rate of 63.9% and a high proportion of cluster C individuals had low platelets (82.3%) and lymphocytes (67.5%) (Table S5). In week 4, class 3 individuals had a mortality rate of 67.5%, and a high proportion of cluster C individuals had low pH (79.2%), platelets (90.0%), and albumin (80.0%) (Table S6). A Sankey diagram depicting the association between the predictors, latent class, and mortality is shown in Fig. 4. Additionally, an interactive Sankey diagram representing the same relationship is available on the website^11^.

### Longitudinal profile

**Figure 5:**
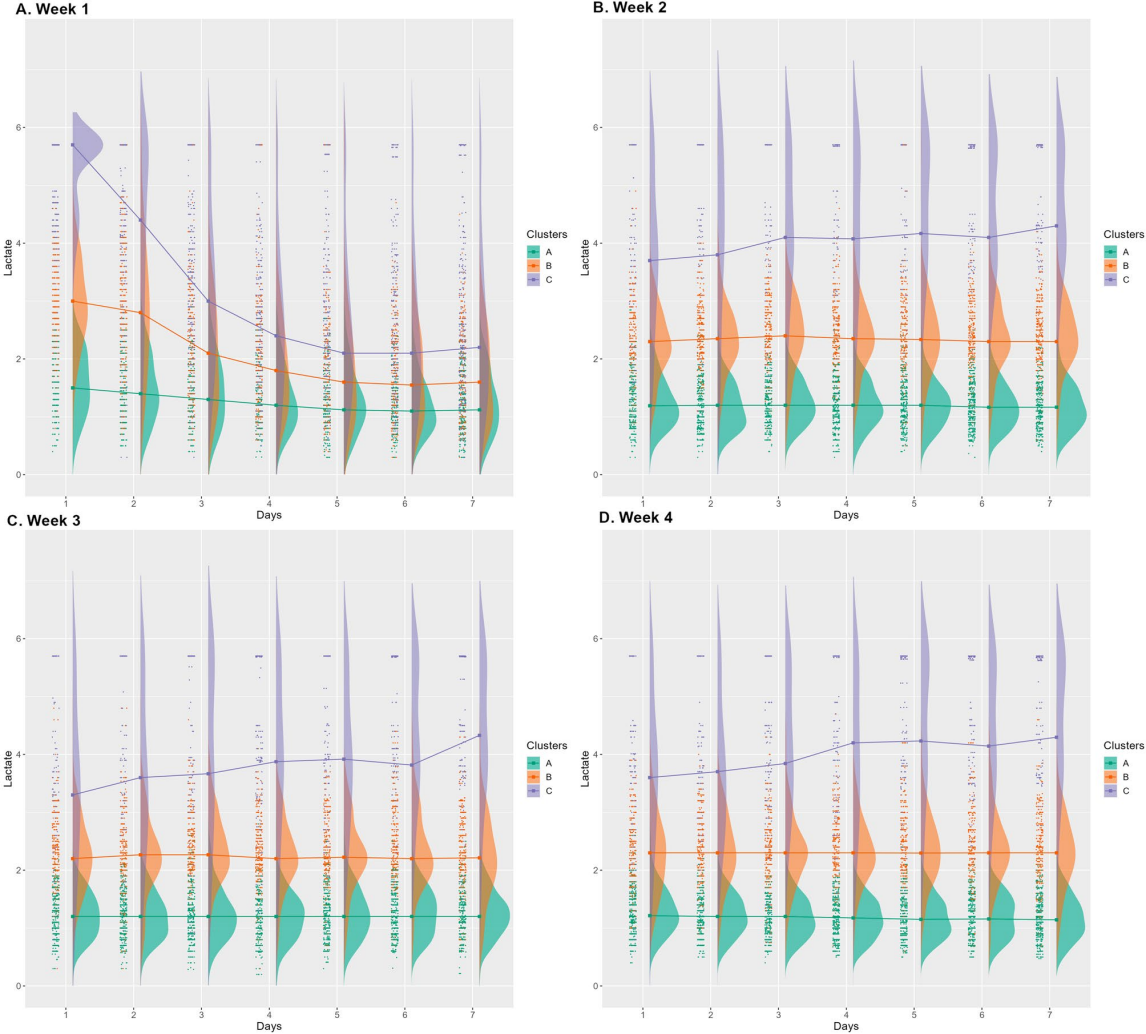
Longitudinal profile of the lactate levels.

**Figure 6:**
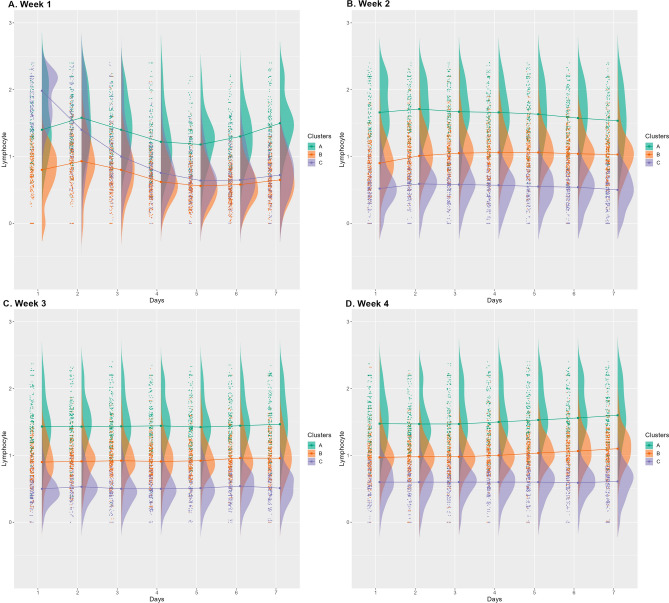
Longitudinal profile of the lymphocyte counts.

**Figure 7:**
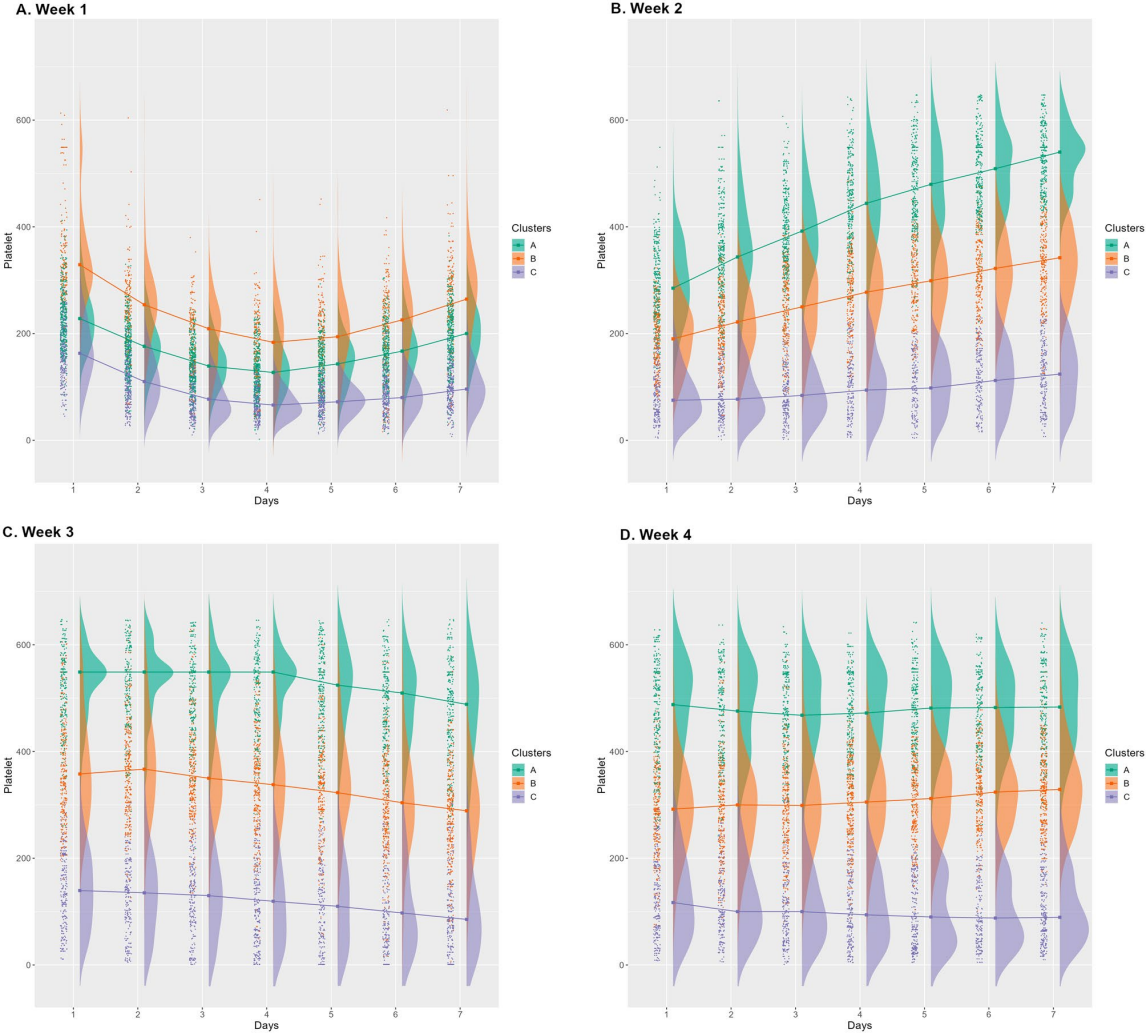
Longitudinal profile of the platelet counts.

Our study found that pH, bicarbonate, RDW, albumin, and lactate displayed similar patterns overall, with slight variations in their levels (Figs. S3–S6). In particular, cluster B in albumin showed a sharp decrease to 3 mg/dL or lower on the first day of week 1, indicating a less favorable outlook compared with cluster A. In addition, lactate levels displayed a sharp decrease from 6 mg/dL in cluster C during week 1 (Fig. 5). The biomarkers of lymphocytes and platelets showed different patterns, with cluster C's high mortality rate in week 1 demonstrating a sharp decrease in the lymphocyte levels (Fig. 6), and cluster B's middle mortality rate showing the highest levels of platelets in week 1 (Fig. 7). The initial clusters, defined using the kmlShape package for these biomarkers, are shown in Figs. S7–S13, and the characteristics and level changes over time are listed in Tables S7–S13.

## Discussion

### Key findings

Our study found that multiple logistic analysis revealed that RDW, bicarbonate, pH, platelets, and lymphocytes were significantly associated with the mortality risk for at least 3 weeks of the study period. The LCA further demonstrated that pH, platelets, lymphocytes, lactate, and albumin were present in the cluster with the highest risk of mortality. In week 1, several biomarkers were significant predictors of mortality risk, with the lowest pH and lactate levels being particularly noteworthy. During this period, AKI was more prevalent than sepsis until day 3, after which sepsis became more prevalent (Fig. 2). This indicates that these biomarkers may be related to both AKI and sepsis. During week 2, pH and lymphocyte count were found to be particularly significant predictors of mortality risk. Lymphocyte and platelet counts were also significant predictors of survival in week 3. This suggests that, during these periods, lymphocytes and platelets, which are markers of infection^12–14^, may have been significant predictors. However, pH, platelet count, and albumin levels were considered important in the fourth week. In particular, the albumin level is an indicator of inflammation^15^ and nutrition. During this period, patients with burns remain susceptible to infections as they are in the process of recovering from burns and undergoing a reduction in nutritional loss due to a decrease in the unhealed skin area.

### Relationship to previous studies

RDW is a known predictor of mortality in critically ill patients and has been found to be higher in the non-survival group of patients with burns at different time points^16^. While decreased RDW has no clinical implications, increased RDW, which reflects large variations in the size of red blood cells, is clinically meaningful^17^. Previous studies have reported the predictive power of RDW for mortality in patients with burns. The RDW reference interval varies with age, sex, race, and the instrument used, with the lower and upper limit being approximately 11.5% and 15%, respectively^17^. In this study, RDW was found to be significant for 3 weeks, and clusters with RDW > 15.5% had higher mortality rates (Table S7).

Platelets are crucial for hemostasis and wound healing; however, they are vulnerable to infection and can contribute to coagulopathy in patients with burns^18^. Recent studies have suggested that platelets play a key role in the pathophysiology of sepsis and are involved in organ dysfunction through their activation in response to inflammatory coagulation reactions and damaged endothelial cells^13^. Platelet count is strongly associated with mortality in patients with burns, with longitudinal changes in platelet count predicting survival^19^. In our study, the median platelet count was below 100 × 10^3^/μL in cluster C, which was associated with a high risk of mortality (Table S10).

Inflammation and the body's immune response to pathogens involve changes in neutrophil and lymphocyte levels^20^ which are typically accompanied by an increase in the neutrophil counts and decrease in the lymphocyte counts; however, the decline in the lymphocyte count may be delayed^21^. Low lymphocyte count has been associated with shorter survival times in sepsis^14^. Lymphopenia, or a low lymphocyte count, is a common feature of sepsis-induced immunosuppression and can impair microbial clearance, increasing the risk of serious infections, which are the leading causes of death in sepsis^14^. In our study, the median lymphocyte count was below 0.6 × 10^3^/μL in weeks 2–4 and was associated with a poor prognosis (Table S11). Albumin reflects the nutritional status, organic function, and physical activity of patients. However, inflammation can decrease albumin production in the liver through the actions of interleukin-1 or tumor necrosis factor^15,22^ contributing to hypoalbuminemia, which is observed in the early stages of sepsis. In our study, the albumin levels were significantly different at weeks 1 and 4, and a higher proportion of cluster C had low albumin levels at week 4 (Table S6). The median albumin levels were < 2.3 g/dL at week 4 (Table S13).

Lactate is a marker of cellular hypoxia and shock and has been linked to mortality in patients with burns^23^. Lower pH and elevated lactate levels are strongly associated with mortality. In the early stages of burns, the changes in these marker levels may be attributed to local or general tissue hypoperfusion due to burn shock or overhydration during fluid resuscitation^24^. Lactate levels and pH are also known predictors of sepsis, which is common in patients with severe burns. Therefore, it is important to consider the potential mechanisms of disease progression when interpreting these markers as they may change over time. In our study, the lactate levels were significant at week 1, and pH was significant in the early periods and was also meaningful according to the LCA. We inferred that the pH and lactate levels were associated with the development of AKI during these periods. The median pH and lactate levels are presented in Tables S9 and S12, respectively.

### Strengths and limitations

In this study, we utilized a novel approach using longitudinal data categorized by k-means clustering algorithms and LCA. This allowed us to identify heterogeneity in the data without the potential for human selection bias, using the most extensive available dataset. However, it is important to note that our study was conducted at a single center in one country, which may have introduced geographical bias and limited the generalizability of our findings to other populations. Future studies with similar designs could validate these findings in other institutions. Additionally, we divided our cohort into four groups based on the time periods, although the clinical meaning of these time periods was not clearly defined and may have introduced a lead-time bias. This division was essential for our primary goal of assessing the trajectories of various biomarkers over time, as the pathophysiology and treatment of burns evolve and can involve fluid resuscitation, hypermetabolic phases, and possible complications, such as AKI, sepsis, and ARDS. Dividing the cohort into four groups allowed the identification of specific patterns of biomarkers. Furthermore, complications, such as sepsis, ARDS, and AKI, developed during the study period, and our focus on predicting mortality rates rather than analyzing the causes of such deaths represents another limitation. These considerations provide the context for our findings and should be considered when interpreting the results.

## Conclusions

In summary, our study found that several biomarkers were significantly associated with the mortality risk in patients with burns. Multiple logistic analyses and LCAs identified pH, lactate, platelets, lymphocytes, and albumin as the markers present in the cluster with the highest risk of mortality. These biomarkers were important to consider in different time periods of the study, with pH and lactate being particularly significant predictors at week 1; pH, platelets, and lymphocytes at week 2; platelets and lymphocytes at week 3; and platelets, lymphocytes, and albumin at week 4. The prevalence of AKI and sepsis also changed over time, with AKI being more prevalent until day 3 in the early stages, and sepsis increasing later. These findings suggest that lactate levels are a better indicator of cellular hypoxia in the early stages of burn shock, whereas platelet and lymphocyte levels are more indicative of infections such as sepsis. Albumin levels are a better indicator of reduced nutritional loss as unhealed burn wounds decrease, while the pH levels reflect the overall condition of the patient throughout the study period. The study used a novel approach of using longitudinal data categorized using k-means clustering algorithms and LCA to avoid human selection bias. The strengths of this study include its longitudinal design and use of advanced data analysis methods. However, it is important to note that our study was conducted at a single center in one country, which may have introduced geographical bias and limited the generalizability of our findings. Future studies with similar designs could validate these findings in other populations.

### Supplementary Information

Supplementary Information.

## Data Availability

The datasets used and/or analyzed during the current study are available from the corresponding author upon reasonable request.

## Abbreviations

LCA: Latent Class Analysis
BICU: Burn Intensive Care Unit
TBSA: Total Body Surface Area
APACHE: Acute Physiology and Chronic Health Evaluation Score
SOFA: Sequential Organ Failure Assessment
AKI: Acute Kidney Injury
AKIN: Acute Kidney Injury Network
AIC: Akaike Information Criteria
BIC: Bayesian Information Criteria
IQR: Interquartile Range
ABSI: Abbreviated Burn Severity Index
WBC: White Blood Cell
RDW: Red cell Distribution Width
BUN: Blood Urea Nitrogen
AST: Aspartate Aminotransferase
ALT: Alanine Aminotransferase
TB: Total Bilirubin
CK: Creatine Kinase
LD: Lactate Dehydrogenase
pCO2: Partial pressure of Carbon Dioxide
pO2: Partial pressure of Oxygen
OR: Odds Ratio
CRRT: Continuous Renal Replacement Therapy
