# Supplementary material for: Evaluating clinical heterogeneity and predicting mortality in severely burned patients through unsupervised clustering and latent class analysis
Source: Sci Rep. 2023 Aug 21;13:13600. doi: 10.1038/s41598-023-40927-7 (PMC10442401; doi:10.1038/s41598-023-40927-7)
Supplement: Supplementary file 1 — Supplementary Information. [file 41598_2023_40927_MOESM1_ESM.docx]

**Supplementary material**

**Utilizing Unsupervised Clustering and Latent Class Analysis to Evaluate Clinical Heterogeneity and Predict Mortality in Severely Burned Patients: A Retrospective Cohort Study"**

Jaechul Yoon1, Dohern Kym1,2*+, Jun Hur1+ Myongjin Kim1, Jongsoo Park1 , Yong-Suk Cho1, Wook Chun1,2, Dogeon Yoon2

1Department of Surgery and Critical Care, Burn Center, Hangang Sacred Heart Hospital, Hallym University Medical Center, 12, Beodeunaru-ro 7-gil, Youngdeungpo-gu, Seoul, Korea, 07247

2Burn Institutes, Hangang Sacred Heart Hospital, Hallym University Medical Center, 12, Beodeunaru-ro 7-gil, Youngdeungpo-gu, Seoul, Korea, 07247

+Dohern Kym and Jun Hur contributed equally in this work as corresponding author.

*Corresponding authors

Department of Surgery and Critical Care, Burn Center, Hangang Sacred Heart Hospital, College of Medicine, Hallym University 12, Beodeunaru-ro 7-gil, Youngdeungpo-gu, Seoul, Korea, 07247

Tel. 82-2-2639-5446, Fax. 82-2-2678-4386, E-mail: dohern@hallym.or.kr

Contents

STROBE Statement—Checklist of items that should be included in reports of cohort studies 4

Fig. S1. Plot showing the missingness of longitudinal data for each time period." 8

Fig. S2. Plot showing the follow-up of enrolled patients during the study periods." 9

Table S1. Comparison of Characteristics between Survivors and Non-Survivors in Four Groups 10

Table S2. Multiple logistic regression for each group 15

Tables. Analyzing the Association between Latent Classes, Mortality, and Predictors 19

Table S3. For Week 1 19

Table S4. For Week 2 21

Table S5. For Week 3 23

Table S6. For Week 4 25

Figures. The longitudinal Profile base on Actual Data. (A. Week 1, B. Week 2, C. Week 3, D. Week 4) 27

Fig. S3. For pH 27

Fig. S4. For bicarbonate 28

Fig. S5. For RDW 29

Fig. S6. For albumin 30

Figures The kmlShape package Generated Longitudinal Plots for Each Cluster 31

Fig. S7. For RDW 31

Fig. S8. For bicarbonate 32

Fig. S9. For pH 33

Fig. S10. For platelet 34

Fig. S11. For lymphocyte 35

Fig. S12. For lactate 36

Fig. S13. For albumin 37

Tables. The Characteristics and levels Change over time in Positive/Negative Group 38

Table S7. For RDW 38

Table S8. For bicarbonate 41

Table S9. For pH 44

Table S10. For platelet 47

Table S11. For lymphocyte 50

Table S12. For lactate 53

Table S13. For albumin 56

# STROBE Statement—Checklist of items that should be included in reports of cohort studies

|  | | | Item No | Recommendation | Checked | |  |
| --- | --- | --- | --- | --- | --- | --- | --- |
| **Title and abstract** | | | 1 | (*a*) Indicate the study’s design with a commonly used term in the title or the abstract | Yes | |  |
|  |  |  |  | (*b*) Provide in the abstract an informative and balanced summary of what was done and what was found | Yes | |  |
| Introduction | | | | | | |  |
| Background/rationale | | | 2 | Explain the scientific background and rationale for the investigation being reported | Yes | |  |
| Objectives | | | 3 | State specific objectives, including any prespecified hypotheses | Yes | |  |
| Methods | | | | | | |  |
| Study design | | | 4 | Present key elements of study design early in the paper | Yes | |  |
| Setting | | | 5 | Describe the setting, locations, and relevant dates, including periods of recruitment, exposure, follow-up, and data collection | Yes | |  |
| Participants | | | 6 | (*a*) Give the eligibility criteria, and the sources and methods of selection of participants. Describe methods of follow-up | Yes | |  |
|  |  |  |  | (*b*) For matched studies, give matching criteria and number of exposed and unexposed | Yes | |  |
| Variables | | | 7 | Clearly define all outcomes, exposures, predictors, potential confounders, and effect modifiers. Give diagnostic criteria, if applicable | Yes | |  |
| Data sources/ measurement | | | 8* | For each variable of interest, give sources of data and details of methods of assessment (measurement). Describe comparability of assessment methods if there is more than one group | Yes | |  |
| Bias | | | 9 | Describe any efforts to address potential sources of bias | Yes | |  |
| Study size | | | 10 | Explain how the study size was arrived at | No | |  |
| Quantitative variables | | | 11 | Explain how quantitative variables were handled in the analyses. If applicable, describe which groupings were chosen and why | Yes | |  |
| Statistical methods | | | 12 | (*a*) Describe all statistical methods, including those used to control for confounding | Yes | |  |
|  |  |  |  | (*b*) Describe any methods used to examine subgroups and interactions | Yes | |  |
|  |  |  |  | (*c*) Explain how missing data were addressed | Yes | |  |
|  |  |  |  | (*d*) If applicable, explain how loss to follow-up was addressed | NA | |  |
|  |  |  |  | (*e*) Describe any sensitivity analyses | NA | |  |
| Results | | | | |  | |  |
| Participants | | | 13* | (a) Report numbers of individuals at each stage of study—eg numbers potentially eligible, examined for eligibility, confirmed eligible, included in the study, completing follow-up, and analysed | Yes | |  |
|  |  |  |  | (b) Give reasons for non-participation at each stage | NA | |  |
|  |  |  |  | (c) Consider use of a flow diagram | Yes | |  |
| Descriptive data | | | 14* | (a) Give characteristics of study participants (eg demographic, clinical, social) and information on exposures and potential confounders | Yes | |  |
|  |  |  |  | (b) Indicate number of participants with missing data for each variable of interest | Yes | |  |
|  |  |  |  | (c) Summarise follow-up time (eg, average and total amount) | NA | |  |
| Outcome data | | | 15* | Report numbers of outcome events or summary measures over time | Yes | |  |
| Main results | 16 | (*a*) Give unadjusted estimates and, if applicable, confounder-adjusted estimates and their precision (eg, 95% confidence interval). Make clear which confounders were adjusted for and why they were included | | | | Yes | |
|  |  | (*b*) Report category boundaries when continuous variables were categorized | | | | Yes | |
|  |  | (*c*) If relevant, consider translating estimates of relative risk into absolute risk for a meaningful time period | | | | Yes | |
| Other analyses | 17 | Report other analyses done—eg analyses of subgroups and interactions, and sensitivity analyses | | | | Yes | |
| Discussion | | | | | | | |
| Key results | 18 | Summarise key results with reference to study objectives | | | | Yes | |
| Limitations | 19 | Discuss limitations of the study, taking into account sources of potential bias or imprecision. Discuss both direction and magnitude of any potential bias | | | | Yes | |
| Interpretation | 20 | Give a cautious overall interpretation of results considering objectives, limitations, multiplicity of analyses, results from similar studies, and other relevant evidence | | | | Yes | |
| Generalisability | 21 | Discuss the generalisability (external validity) of the study results | | | | Yes | |
| Other information | | | | | | | |
| Funding | 22 | Give the source of funding and the role of the funders for the present study and, if applicable, for the original study on which the present article is based | | | | Yes | |

*Give information separately for exposed and unexposed groups.

**Note:** An Explanation and Elaboration article discusses each checklist item and gives methodological background and published examples of transparent reporting. The STROBE checklist is best used in conjunction with this article (freely available on the Web sites of PLoS Medicine at http://www.plosmedicine.org/, Annals of Internal Medicine at http://www.annals.org/, and Epidemiology at http://www.epidem.com/). Information on the STROBE Initiative is available at http://www.strobe-statement.org.

## Fig. S1. Plot showing the missingness of longitudinal data for each time period."


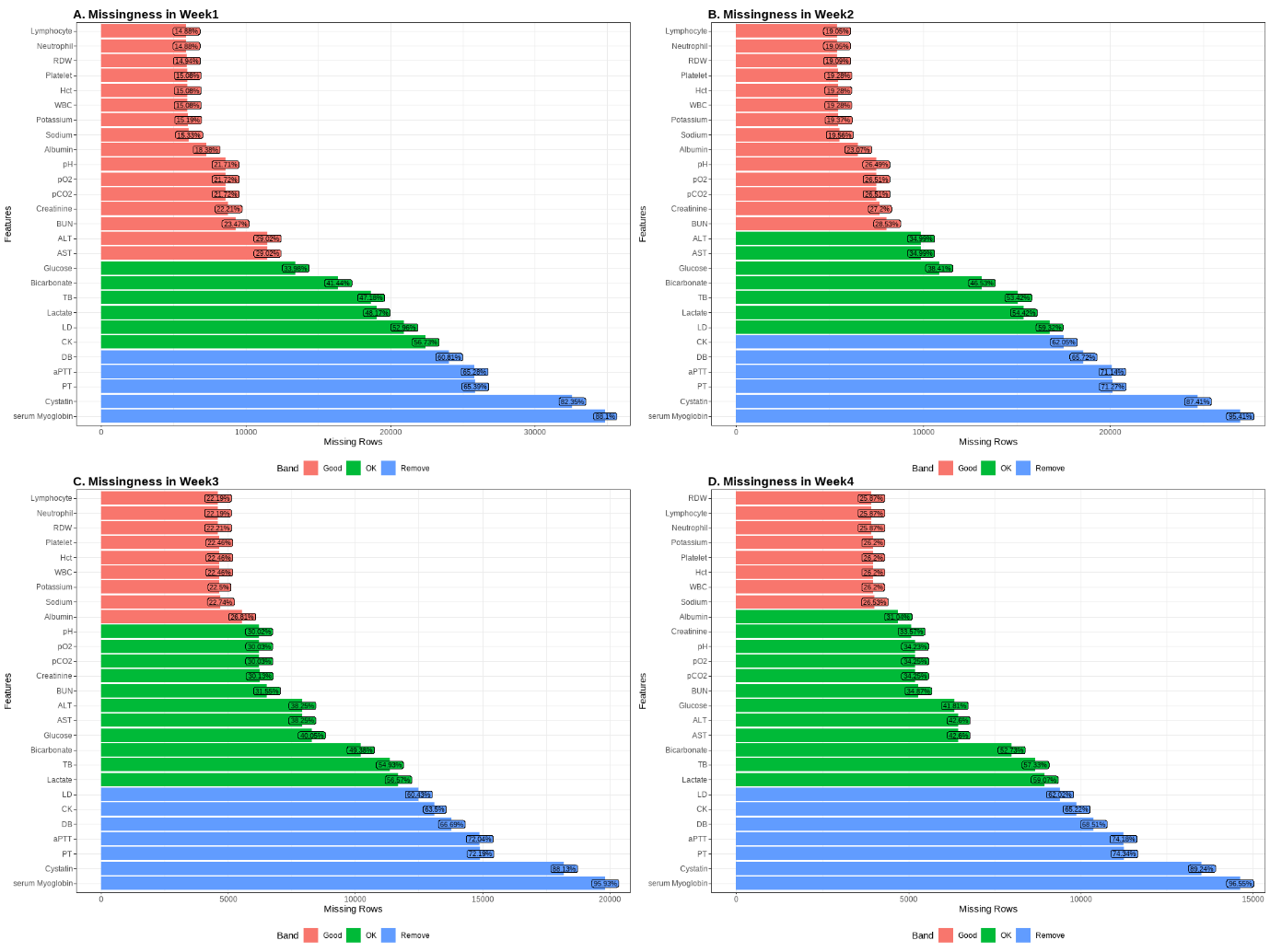


## Fig. S2. Plot showing the follow-up of enrolled patients during the study periods."


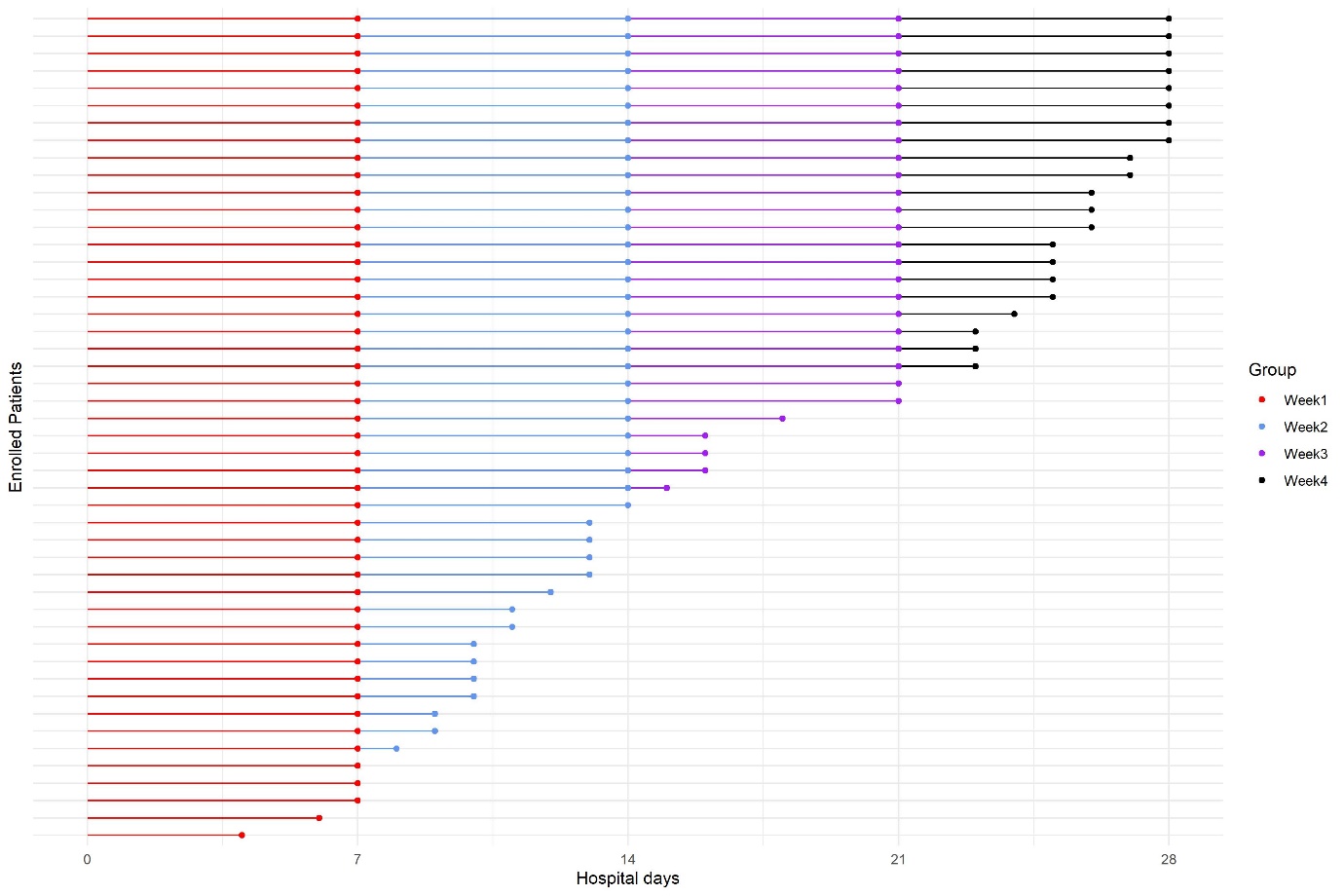


## Table S1. Comparison of Characteristics between Survivors and Non-Survivors in Four Groups

|  | | Week1 | | | | Week2 | | | | Week3 | | | | Week4 | | | |
| --- | --- | --- | --- | --- | --- | --- | --- | --- | --- | --- | --- | --- | --- | --- | --- | --- | --- |
| Group | Variables | Overall, N = 1,727 | Survivors, N = 1338 (77.5%) | Non_Survivors, N = 389 (22.5%) | p-value | Overall, N = 1,268 | Survivors, N = 964 (76.0%) | Non_Survivors, N = 304 (24.0%) | p-value | Overall, N = 882 | Survivors, N = 694 (78.7%) | Non_Survivors, N = 188 (21.3%) | p-value | Overall, N = 662 | Survivors, N = 550 (83.1%) | Non_Survivors, N = 112 (16.9%) | p-value |
| Demographics | Patient Age |  |  |  | <0.001 |  |  |  | <0.001 |  |  |  | <0.001 |  |  |  | <0.001 |
|  | Median [IQR] | 50 [40, 60] | 49 [39, 58] | 55 [45, 66] |  | 51 [41, 61] | 49 [40, 59] | 54 [45, 66] |  | 51 [41, 61] | 50 [41, 59] | 53 [45, 65] |  | 52 [42, 62] | 51 [41, 60] | 55 [46, 70] |  |
|  | Sex |  |  |  | 0.825 |  |  |  | 0.682 |  |  |  | 0.545 |  |  |  | 0.900 |
|  | Male | 1,402 (81.2%) | 1,088 (81.3%) | 314 (80.7%) |  | 1,013 (79.9%) | 767 (79.6%) | 246 (80.9%) |  | 696 (78.9%) | 551 (79.4%) | 145 (77.1%) |  | 519 (78.4%) | 432 (78.5%) | 87 (77.7%) |  |
|  | Female | 325 (18.8%) | 250 (18.7%) | 75 (19.3%) |  | 255 (20.1%) | 197 (20.4%) | 58 (19.1%) |  | 186 (21.1%) | 143 (20.6%) | 43 (22.9%) |  | 143 (21.6%) | 118 (21.5%) | 25 (22.3%) |  |
|  | Type |  |  |  | <0.001 |  |  |  | <0.001 |  |  |  | <0.001 |  |  |  | 0.070 |
|  | FB | 1,274 (73.8%) | 934 (69.8%) | 340 (87.4%) |  | 967 (76.3%) | 707 (73.3%) | 260 (85.5%) |  | 683 (77.4%) | 521 (75.1%) | 162 (86.2%) |  | 511 (77.2%) | 415 (75.5%) | 96 (85.7%) |  |
|  | SB | 144 (8.3%) | 117 (8.7%) | 27 (6.9%) |  | 110 (8.7%) | 84 (8.7%) | 26 (8.6%) |  | 78 (8.8%) | 63 (9.1%) | 15 (8.0%) |  | 60 (9.1%) | 51 (9.3%) | 9 (8.0%) |  |
|  | EB | 234 (13.5%) | 223 (16.7%) | 11 (2.8%) |  | 132 (10.4%) | 124 (12.9%) | 8 (2.6%) |  | 78 (8.8%) | 75 (10.8%) | 3 (1.6%) |  | 59 (8.9%) | 56 (10.2%) | 3 (2.7%) |  |
|  | ChB | 23 (1.3%) | 20 (1.5%) | 3 (0.8%) |  | 18 (1.4%) | 16 (1.7%) | 2 (0.7%) |  | 13 (1.5%) | 11 (1.6%) | 2 (1.1%) |  | 8 (1.2%) | 7 (1.3%) | 1 (0.9%) |  |
|  | CoB | 52 (3.0%) | 44 (3.3%) | 8 (2.1%) |  | 41 (3.2%) | 33 (3.4%) | 8 (2.6%) |  | 30 (3.4%) | 24 (3.5%) | 6 (3.2%) |  | 24 (3.6%) | 21 (3.8%) | 3 (2.7%) |  |
|  | TBSA |  |  |  | <0.001 |  |  |  | <0.001 |  |  |  | <0.001 |  |  |  | <0.001 |
|  | Median [IQR] | 30 [19, 49] | 25 [15, 38] | 62 [40, 83] |  | 34 [22, 52] | 30 [20, 42] | 62 [40, 80] |  | 38 [24, 54] | 34 [21, 47] | 60 [40, 75] |  | 38 [24, 54] | 36 [23, 50] | 54 [33, 65] |  |
|  | Inhalation | 764 (44.2%) | 528 (39.5%) | 236 (60.7%) | <0.001 | 575 (45.3%) | 407 (42.2%) | 168 (55.3%) | <0.001 | 397 (45.0%) | 306 (44.1%) | 91 (48.4%) | 0.321 | 309 (46.7%) | 252 (45.8%) | 57 (50.9%) | 0.350 |
|  | LOICU |  |  |  | 0.026 |  |  |  | <0.001 |  |  |  | <0.001 |  |  |  | <0.001 |
|  | Median [IQR] | 15 [7, 30] | 16 [7, 34] | 14 [9, 23] |  | 23 [13, 36] | 25 [13, 40] | 18 [12, 25] |  | 30 [22, 43] | 33 [23, 47] | 24 [19, 30] |  | 36 [27, 49] | 37 [28, 55] | 29 [25, 35] |  |
| Intervention | CRRT apply | 446 (25.8%) | 122 (31.4%) | 324 (24.2%) | 0.006 | 90 (7.1%) | 66 (21.7%) | 24 (2.5%) | <0.001 | 51 (5.8%) | 27 (14.4%) | 24 (3.5%) | <0.001 | 44 (6.6%) | 18 (16.1%) | 26 (4.7%) | <0.001 |
|  | Ventilator apply | 1,178 (68.2%) | 380 (97.7%) | 798 (59.6%) | <0.001 | 773 (61.0%) | 295 (97.0%) | 478 (49.6%) | <0.001 | 590 (66.9%) | 179 (95.2%) | 411 (59.2%) | <0.001 | 464 (70.1%) | 107 (95.5%) | 357 (64.9%) | <0.001 |
| Severity Scores | ABSI |  |  |  | <0.001 |  |  |  | <0.001 |  |  |  | <0.001 |  |  |  | <0.001 |
|  | Median [IQR] | 8 [6, 10] | 7 [6, 9] | 12 [10, 13] |  | 9 [7, 10] | 8 [7, 9] | 12 [10, 13] |  | 9 [7, 10] | 8 [7, 10] | 11 [9, 13] |  | 9 [8, 10] | 9 [7, 10] | 11 [9, 12] |  |
|  | rBaux |  |  |  | <0.001 |  |  |  | <0.001 |  |  |  | <0.001 |  |  |  | <0.001 |
|  | Median [IQR] | 91 [72, 111] | 83 [68, 98] | 128 [108, 146] |  | 96 [79, 115] | 89 [74, 102] | 124 [108, 142] |  | 98 [82, 115] | 94 [79, 108] | 119 [106, 134] |  | 99 [84, 115] | 96 [82, 110] | 117 [105, 130] |  |
|  | Hangang |  |  |  | <0.001 |  |  |  | <0.001 |  |  |  | <0.001 |  |  |  | <0.001 |
|  | Median [IQR] | 132 [121, 147] | 127 [118, 137] | 160 [149, 174] |  | 123 [112, 136] | 118 [110, 128] | 145 [134, 158] |  | 123 [112, 135] | 119 [111, 129] | 142 [130, 155] |  | 124 [114, 136] | 121 [113, 131] | 144 [132, 154] |  |
|  | APACHE IV |  |  |  | <0.001 |  |  |  | <0.001 |  |  |  | <0.001 |  |  |  | <0.001 |
|  | Median [IQR] | 37 [23, 57] | 32 [20, 48] | 58 [44, 78] |  | 37 [26, 52] | 33 [24, 45] | 53 [39, 67] |  | 37 [28, 54] | 35 [26, 46] | 58 [44, 73] |  | 40 [29, 56] | 36 [27, 49] | 65 [52, 84] |  |
|  | SOFA |  |  |  | <0.001 |  |  |  | <0.001 |  |  |  | <0.001 |  |  |  | <0.001 |
|  | Median [IQR] | 3 [2, 5] | 3 [1, 4] | 5 [3, 7] |  | 3 [2, 5] | 3 [1, 4] | 6 [4, 8] |  | 3 [2, 5] | 3 [1, 4] | 5 [4, 8] |  | 3 [1, 5] | 3 [1, 4] | 7 [5, 10] |  |
| Comobidities | Hypertension | 285 (16.5%) | 198 (14.8%) | 87 (22.4%) | <0.001 | 222 (17.5%) | 154 (16.0%) | 68 (22.4%) | 0.011 | 156 (17.7%) | 118 (17.0%) | 38 (20.2%) | 0.306 | 123 (18.6%) | 96 (17.5%) | 27 (24.1%) | 0.099 |
|  | Diabetes Mellitus | 130 (7.5%) | 85 (6.4%) | 45 (11.6%) | <0.001 | 97 (7.6%) | 63 (6.5%) | 34 (11.2%) | 0.008 | 63 (7.1%) | 45 (6.5%) | 18 (9.6%) | 0.144 | 51 (7.7%) | 38 (6.9%) | 13 (11.6%) | 0.089 |
|  | Tuberculosis | 26 (1.5%) | 18 (1.3%) | 8 (2.1%) | 0.311 | 22 (1.7%) | 16 (1.7%) | 6 (2.0%) | 0.715 | 16 (1.8%) | 13 (1.9%) | 3 (1.6%) | >0.999 | 14 (2.1%) | 11 (2.0%) | 3 (2.7%) | 0.716 |
|  | Hepatobiliary | 34 (2.0%) | 28 (2.1%) | 6 (1.5%) | 0.492 | 23 (1.8%) | 18 (1.9%) | 5 (1.6%) | 0.800 | 13 (1.5%) | 11 (1.6%) | 2 (1.1%) | >0.999 | 11 (1.7%) | 9 (1.6%) | 2 (1.8%) | >0.999 |
|  | Cardiovascular | 39 (2.3%) | 31 (2.3%) | 8 (2.1%) | 0.761 | 30 (2.4%) | 23 (2.4%) | 7 (2.3%) | 0.934 | 22 (2.5%) | 19 (2.7%) | 3 (1.6%) | 0.597 | 14 (2.1%) | 13 (2.4%) | 1 (0.9%) | 0.484 |
|  | CVA | 23 (1.3%) | 17 (1.3%) | 6 (1.5%) | 0.681 | 22 (1.7%) | 17 (1.8%) | 5 (1.6%) | 0.890 | 19 (2.2%) | 16 (2.3%) | 3 (1.6%) | 0.778 | 14 (2.1%) | 11 (2.0%) | 3 (2.7%) | 0.716 |
|  | Cancer | 41 (2.4%) | 28 (2.1%) | 13 (3.3%) | 0.154 | 28 (2.2%) | 18 (1.9%) | 10 (3.3%) | 0.141 | 21 (2.4%) | 12 (1.7%) | 9 (4.8%) | 0.026 | 15 (2.3%) | 9 (1.6%) | 6 (5.4%) | 0.028 |
|  | Hyperlipidemia | 45 (2.6%) | 35 (2.6%) | 10 (2.6%) | 0.961 | 35 (2.8%) | 27 (2.8%) | 8 (2.6%) | 0.875 | 23 (2.6%) | 18 (2.6%) | 5 (2.7%) | >0.999 | 16 (2.4%) | 13 (2.4%) | 3 (2.7%) | 0.742 |
|  | Other | 432 (25.0%) | 343 (25.6%) | 89 (22.9%) | 0.269 | 329 (25.9%) | 263 (27.3%) | 66 (21.7%) | 0.053 | 234 (26.5%) | 192 (27.7%) | 42 (22.3%) | 0.142 | 183 (27.6%) | 158 (28.7%) | 25 (22.3%) | 0.167 |

## Table S2. Multiple logistic regression for each group

|  | Week1 | | Week2 | | Week3 | | Week4 | |
| --- | --- | --- | --- | --- | --- | --- | --- | --- |
| Characteristic | OR (95% CI)^1^ | p-value | OR (95% CI)^1^ | p-value | OR (95% CI)^1^ | p-value | OR (95% CI)^1^ | p-value |
| Age | 1.056 (1.040, 1.073) | **<0.001** | 1.048 (1.031, 1.067) | **<0.001** | 1.027 (1.007, 1.048) | **0.006** | 1.035 (1.011, 1.061) | **0.004** |
| TBSA | 1.059 (1.047, 1.071) | **<0.001** | 1.043 (1.030, 1.057) | **<0.001** | 1.030 (1.015, 1.046) | **<0.001** | 1.027 (1.007, 1.048) | **0.008** |
| Apply of Ventilator |  | **<0.001** |  | **0.003** |  | 0.073 |  |  |
| Yes | 5.708 (2.723, 13.33) |  | 3.284 (1.469, 8.113) |  | 2.176 (0.933, 5.508) |  |  |  |
| Apply of CRRT |  | **0.024** |  |  |  |  |  |  |
| Yes | 2.011 (1.096, 3.705) |  |  |  |  |  |  |  |
| RDW |  | **<0.001** |  | **<0.001** |  | **0.002** |  | **0.002** |
| B | 2.958 (1.635, 5.525) |  | 5.624 (1.806, 23.73) |  | 3.739 (1.399, 12.06) |  | 1.322 (0.461, 4.441) |  |
| C | 5.714 (2.841, 11.78) |  | 8.160 (2.539, 34.90) |  | 6.216 (2.143, 21.16) |  | 4.521 (1.435, 16.48) |  |
| Bicarbonate |  | **<0.001** |  | **<0.001** |  | **0.039** |  |  |
| B | 0.740 (0.430, 1.275) |  | 2.317 (1.226, 4.491) |  | 1.211 (0.541, 2.773) |  |  |  |
| C | 1.818 (0.997, 3.336) |  | 3.867 (1.972, 7.794) |  | 2.511 (1.035, 6.217) |  |  |  |
| pH |  | **0.004** |  | **<0.001** |  | 0.115 |  | **0.004** |
| B | 0.739 (0.387, 1.430) |  | 1.735 (0.833, 3.820) |  | 1.169 (0.507, 2.808) |  | 1.705 (0.640, 4.990) |  |
| C | 1.594 (0.814, 3.177) |  | 6.667 (2.903, 16.12) |  | 2.393 (0.869, 6.781) |  | 6.772 (1.901, 25.63) |  |
| Platelet |  |  |  | **<0.001** |  | **0.002** |  | **<0.001** |
| B |  |  | 1.643 (0.798, 3.577) |  | 2.792 (1.038, 8.954) |  | 1.503 (0.463, 5.915) |  |
| C |  |  | 4.430 (2.103, 9.850) |  | 5.748 (2.052, 19.00) |  | 6.126 (1.946, 23.90) |  |
| Lymphocyte |  | 0.078 |  | **0.014** |  | **0.007** |  | **<0.001** |
| B | 2.238 (1.100, 4.764) |  | 0.581 (0.263, 1.303) |  | 0.981 (0.417, 2.393) |  | 1.236 (0.393, 4.363) |  |
| C | 1.827 (0.888, 3.909) |  | 1.210 (0.552, 2.695) |  | 2.387 (1.006, 5.893) |  | 4.508 (1.510, 15.67) |  |
| Albumin |  | **0.003** |  |  |  |  |  | **0.026** |
| B | 1.562 (0.506, 6.011) |  |  |  |  |  | 1.835 (0.304, 35.87) |  |
| C | 3.154 (0.972, 12.58) |  |  |  |  |  | 4.852 (0.744, 97.80) |  |
| Creatinine |  | 0.104 |  | **<0.001** |  | **0.047** |  |  |
| B | 1.152 (0.713, 1.865) |  | 0.946 (0.550, 1.604) |  | 2.017 (1.077, 3.764) |  |  |  |
| C | 1.973 (1.030, 3.783) |  | 3.680 (1.807, 7.668) |  | 2.307 (1.014, 5.260) |  |  |  |
| pCO2 |  |  |  |  |  | **0.005** |  | **0.001** |
| B |  |  |  |  | 2.033 (0.965, 4.390) |  | 0.462 (0.198, 1.065) |  |
| C |  |  |  |  | 4.903 (1.874, 13.17) |  | 2.187 (0.749, 6.323) |  |
| Lactate |  | **0.029** |  |  |  |  |  |  |
| B | 2.043 (0.989, 4.487) |  |  |  |  |  |  |  |
| C | 2.652 (1.280, 5.827) |  |  |  |  |  |  |  |
| AST |  | **0.003** |  |  |  |  |  |  |
| B | 1.726 (1.068, 2.797) |  |  |  |  |  |  |  |
| C | 2.414 (1.446, 4.054) |  |  |  |  |  |  |  |
| BUN |  | **<0.001** |  |  |  |  |  |  |
| B | 0.844 (0.516, 1.371) |  |  |  |  |  |  |  |
| C | 2.385 (1.328, 4.303) |  |  |  |  |  |  |  |
| Glucose |  | **0.027** |  |  |  |  |  |  |
| B | 1.243 (0.729, 2.139) |  |  |  |  |  |  |  |
| C | 2.018 (1.148, 3.575) |  |  |  |  |  |  |  |
| pO2 |  | 0.122 |  |  |  | **0.028** |  |  |
| B | 1.499 (0.924, 2.439) |  |  |  | 2.468 (1.249, 4.952) |  |  |  |
| C | 0.901 (0.564, 1.438) |  |  |  | 1.582 (0.828, 3.038) |  |  |  |
| WBC |  |  |  |  |  | **0.026** |  |  |
| B |  |  |  |  | 1.824 (0.891, 3.762) |  |  |  |
| C |  |  |  |  | 2.269 (1.229, 4.224) |  |  |  |
| Potassium |  |  |  |  |  |  |  | **0.030** |
| B |  |  |  |  |  |  | 0.642 (0.278, 1.473) |  |
| C |  |  |  |  |  |  | 1.875 (0.757, 4.754) |  |
| ^1^OR = Odds Ratio, CI = Confidence Interval | | | | | | | | |

# Tables. Analyzing the Association between Latent Classes, Mortality, and Predictors

## Table S3. For Week 1

| **Variables** | **Class** **1**, N = 599 (34.7%) | **Class** **2**, N = 717 (41.5%) | **Class** **3**, N = 411 (23.8%) | **p-value** |
| --- | --- | --- | --- | --- |
| Mortality | 4 (0.7%) | 88 (12.3%) | 297 (72.3%) | <0.001 |
| Age |  |  |  | <0.001 |
| Median [IQR] | 47 [37, 56] | 52 [42, 60] | 53 [43, 63] |  |
| TBSA |  |  |  | <0.001 |
| Median [IQR] | 20 [10, 30] | 31 [20, 44] | 60 [40, 80] |  |
| Inhalation | 205 (34.2%) | 302 (42.1%) | 257 (62.5%) | <0.001 |
| Apply of CRRT | 237 (39.6%) | 84 (11.7%) | 125 (30.4%) | <0.001 |
| Apply of Ventilator | 309 (51.6%) | 473 (66.0%) | 396 (96.4%) | <0.001 |
| RDW |  |  |  | <0.001 |
| A | 506 (84.5%) | 294 (41.0%) | 2 (0.5%) |  |
| B | 74 (12.4%) | 354 (49.4%) | 263 (64.0%) |  |
| C | 19 (3.2%) | 69 (9.6%) | 146 (35.5%) |  |
| Albumin |  |  |  | <0.001 |
| A | 261 (43.6%) | 45 (6.3%) | 0 (0.0%) |  |
| B | 308 (51.4%) | 560 (78.1%) | 158 (38.4%) |  |
| C | 30 (5.0%) | 112 (15.6%) | 253 (61.6%) |  |
| Lactate |  |  |  | <0.001 |
| A | 358 (59.8%) | 71 (9.9%) | 12 (2.9%) |  |
| B | 197 (32.9%) | 375 (52.3%) | 47 (11.4%) |  |
| C | 44 (7.3%) | 271 (37.8%) | 352 (85.6%) |  |
| AST |  |  |  | <0.001 |
| A | 365 (60.9%) | 340 (47.4%) | 69 (16.8%) |  |
| B | 103 (17.2%) | 208 (29.0%) | 129 (31.4%) |  |
| C | 131 (21.9%) | 169 (23.6%) | 213 (51.8%) |  |
| BUN |  |  |  | <0.001 |
| A | 498 (83.1%) | 445 (62.1%) | 99 (24.1%) |  |
| B | 94 (15.7%) | 212 (29.6%) | 138 (33.6%) |  |
| C | 7 (1.2%) | 60 (8.4%) | 174 (42.3%) |  |
| Glucose |  |  |  | <0.001 |
| A | 461 (77.0%) | 247 (34.4%) | 27 (6.6%) |  |
| B | 113 (18.9%) | 334 (46.6%) | 156 (38.0%) |  |
| C | 25 (4.2%) | 136 (19.0%) | 228 (55.5%) |  |
| Bicarbonate |  |  |  | <0.001 |
| A | 430 (71.8%) | 201 (28.0%) | 22 (5.4%) |  |
| B | 143 (23.9%) | 380 (53.0%) | 235 (57.2%) |  |
| C | 26 (4.3%) | 136 (19.0%) | 154 (37.5%) |  |
| pH |  |  |  | <0.001 |
| A | 400 (66.8%) | 160 (22.3%) | 5 (1.2%) |  |
| B | 194 (32.4%) | 422 (58.9%) | 58 (14.1%) |  |
| C | 5 (0.8%) | 135 (18.8%) | 348 (84.7%) |  |

## Table S4. For Week 2

| **Variables** | **Class** **1**, N = 633 (49.9%) | **Class** **2**, N = 355 (28.0%) | **Class** **3**, N = 280 (22.1%) | **p-value** |
| --- | --- | --- | --- | --- |
| Mortality | 6 (0.9%) | 68 (19.2%) | 230 (82.1%) | <0.001 |
| Age |  |  |  | <0.001 |
| Median [IQR] | 48 [39, 58] | 52 [42, 62] | 54 [45, 65] |  |
| TBSA |  |  |  | <0.001 |
| Median [IQR] | 27 [19, 38] | 40 [24, 60] | 62 [40, 81] |  |
| Inhalation | 248 (39.2%) | 167 (47.0%) | 160 (57.1%) | <0.001 |
| Apply of CRRT | 1 (0.2%) | 10 (2.8%) | 79 (28.2%) | <0.001 |
| Apply of Ventilator | 230 (36.3%) | 271 (76.3%) | 272 (97.1%) | <0.001 |
| RDW |  |  |  | <0.001 |
| A | 421 (66.5%) | 9 (2.5%) | 6 (2.1%) |  |
| B | 195 (30.8%) | 233 (65.6%) | 89 (31.8%) |  |
| C | 17 (2.7%) | 113 (31.8%) | 185 (66.1%) |  |
| pH |  |  |  | <0.001 |
| A | 469 (74.1%) | 60 (16.9%) | 0 (0.0%) |  |
| B | 162 (25.6%) | 261 (73.5%) | 43 (15.4%) |  |
| C | 2 (0.3%) | 34 (9.6%) | 237 (84.6%) |  |
| Platelet |  |  |  | <0.001 |
| A | 403 (63.7%) | 90 (25.4%) | 0 (0.0%) |  |
| B | 229 (36.2%) | 172 (48.5%) | 32 (11.4%) |  |
| C | 1 (0.2%) | 93 (26.2%) | 248 (88.6%) |  |
| Bicarbonate |  |  |  | <0.001 |
| A | 288 (45.5%) | 70 (19.7%) | 35 (12.5%) |  |
| B | 265 (41.9%) | 169 (47.6%) | 110 (39.3%) |  |
| C | 80 (12.6%) | 116 (32.7%) | 135 (48.2%) |  |
| Creatinine |  |  |  | <0.001 |
| A | 416 (65.7%) | 265 (74.6%) | 18 (6.4%) |  |
| B | 210 (33.2%) | 80 (22.5%) | 115 (41.1%) |  |
| C | 7 (1.1%) | 10 (2.8%) | 147 (52.5%) |  |
| Lymphocyte |  |  |  | <0.001 |
| A | 239 (37.8%) | 28 (7.9%) | 16 (5.7%) |  |
| B | 310 (49.0%) | 187 (52.7%) | 57 (20.4%) |  |
| C | 84 (13.3%) | 140 (39.4%) | 207 (73.9%) |  |

## Table S5. For Week 3

| **Variables** | **Class** **1**, N = 229 (26.0%) | **Class** **2**, N = 404 (45.8%) | **Class** **3**, N = 249 (28.2%) | **p-value** |
| --- | --- | --- | --- | --- |
| Mortality | 4 (1.7%) | 25 (6.2%) | 159 (63.9%) | <0.001 |
| Age |  |  |  | <0.001 |
| Median [IQR] | 49 [41, 58] | 49 [41, 58] | 54 [45, 66] |  |
| TBSA |  |  |  | <0.001 |
| Median [IQR] | 30 [19, 42] | 37 [25, 50] | 52 [32, 70] |  |
| Inhalation | 109 (47.6%) | 173 (42.8%) | 115 (46.2%) | 0.463 |
| Apply of CRRT | 2 (0.9%) | 5 (1.2%) | 44 (17.7%) | <0.001 |
| Apply of Ventilator | 112 (48.9%) | 245 (60.6%) | 233 (93.6%) | <0.001 |
| RDW |  |  |  | <0.001 |
| A | 136 (59.4%) | 156 (38.6%) | 4 (1.6%) |  |
| B | 77 (33.6%) | 218 (54.0%) | 122 (49.0%) |  |
| C | 16 (7.0%) | 30 (7.4%) | 123 (49.4%) |  |
| Platelet |  |  |  | <0.001 |
| A | 146 (63.8%) | 164 (40.6%) | 0 (0.0%) |  |
| B | 73 (31.9%) | 216 (53.5%) | 44 (17.7%) |  |
| C | 10 (4.4%) | 24 (5.9%) | 205 (82.3%) |  |
| pCO2 |  |  |  | <0.001 |
| A | 36 (15.7%) | 244 (60.4%) | 28 (11.2%) |  |
| B | 143 (62.4%) | 152 (37.6%) | 90 (36.1%) |  |
| C | 50 (21.8%) | 8 (2.0%) | 131 (52.6%) |  |
| Bicarbonate |  |  |  | <0.001 |
| A | 217 (94.8%) | 0 (0.0%) | 30 (12.0%) |  |
| B | 12 (5.2%) | 290 (71.8%) | 95 (38.2%) |  |
| C | 0 (0.0%) | 114 (28.2%) | 124 (49.8%) |  |
| pO2 |  |  |  | <0.001 |
| A | 80 (34.9%) | 181 (44.8%) | 107 (43.0%) |  |
| B | 39 (17.0%) | 132 (32.7%) | 54 (21.7%) |  |
| C | 110 (48.0%) | 91 (22.5%) | 88 (35.3%) |  |
| Lymphocyte |  |  |  | <0.001 |
| A | 104 (45.4%) | 127 (31.4%) | 10 (4.0%) |  |
| B | 102 (44.5%) | 211 (52.2%) | 71 (28.5%) |  |
| C | 23 (10.0%) | 66 (16.3%) | 168 (67.5%) |  |
| Creatinine |  |  |  | <0.001 |
| A | 201 (87.8%) | 330 (81.7%) | 63 (25.3%) |  |
| B | 25 (10.9%) | 67 (16.6%) | 107 (43.0%) |  |
| C | 3 (1.3%) | 7 (1.7%) | 79 (31.7%) |  |
| WBC |  |  |  | <0.001 |
| A | 92 (40.2%) | 190 (47.0%) | 76 (30.5%) |  |
| B | 95 (41.5%) | 124 (30.7%) | 52 (20.9%) |  |
| C | 42 (18.3%) | 90 (22.3%) | 121 (48.6%) |  |

## Table S6. For Week 4

| **Variables** | **Class** **1**, N = 234 (35.3%) | **Class** **2**, N = 308 (46.5%) | **Class** **3**, N = 120 (18.1%) | **p-value** |
| --- | --- | --- | --- | --- |
| Mortality | 2 (0.9%) | 29 (9.4%) | 81 (67.5%) | <0.001 |
| Age |  |  |  | <0.001 |
| Median [IQR] | 48 [40, 56] | 53 [43, 64] | 56 [46, 69] |  |
| TBSA |  |  |  | <0.001 |
| Median [IQR] | 35 [20, 45] | 38 [25, 52] | 56 [32, 68] |  |
| Inhalation | 111 (47.4%) | 133 (43.2%) | 65 (54.2%) | 0.125 |
| Apply of CRRT | 2 (0.9%) | 20 (6.5%) | 22 (18.3%) | <0.001 |
| Apply of Ventilator | 127 (54.3%) | 223 (72.4%) | 114 (95.0%) | <0.001 |
| pH |  |  |  | <0.001 |
| A | 98 (41.9%) | 120 (39.0%) | 0 (0.0%) |  |
| B | 134 (57.3%) | 185 (60.1%) | 25 (20.8%) |  |
| C | 2 (0.9%) | 3 (1.0%) | 95 (79.2%) |  |
| Platelet |  |  |  | <0.001 |
| A | 170 (72.6%) | 32 (10.4%) | 4 (3.3%) |  |
| B | 64 (27.4%) | 201 (65.3%) | 8 (6.7%) |  |
| C | 0 (0.0%) | 75 (24.4%) | 108 (90.0%) |  |
| Albumin |  |  |  | <0.001 |
| A | 99 (42.3%) | 11 (3.6%) | 1 (0.8%) |  |
| B | 127 (54.3%) | 195 (63.3%) | 23 (19.2%) |  |
| C | 8 (3.4%) | 102 (33.1%) | 96 (80.0%) |  |
| RDW |  |  |  | <0.001 |
| A | 138 (59.0%) | 72 (23.4%) | 2 (1.7%) |  |
| B | 85 (36.3%) | 180 (58.4%) | 54 (45.0%) |  |
| C | 11 (4.7%) | 56 (18.2%) | 64 (53.3%) |  |
| Lymphocyte |  |  |  | <0.001 |
| A | 127 (54.3%) | 39 (12.7%) | 7 (5.8%) |  |
| B | 97 (41.5%) | 151 (49.0%) | 36 (30.0%) |  |
| C | 10 (4.3%) | 118 (38.3%) | 77 (64.2%) |  |
| pCO2 |  |  |  | <0.001 |
| A | 71 (30.3%) | 172 (55.8%) | 1 (0.8%) |  |
| B | 135 (57.7%) | 121 (39.3%) | 48 (40.0%) |  |
| C | 28 (12.0%) | 15 (4.9%) | 71 (59.2%) |  |
| Potassium |  |  |  | <0.001 |
| A | 27 (11.5%) | 118 (38.3%) | 15 (12.5%) |  |
| B | 87 (37.2%) | 137 (44.5%) | 64 (53.3%) |  |
| C | 120 (51.3%) | 53 (17.2%) | 41 (34.2%) |  |

# Figures. The longitudinal Profile base on Actual Data. (A. Week 1, B. Week 2, C. Week 3, D. Week 4)

## Fig. S3. For pH


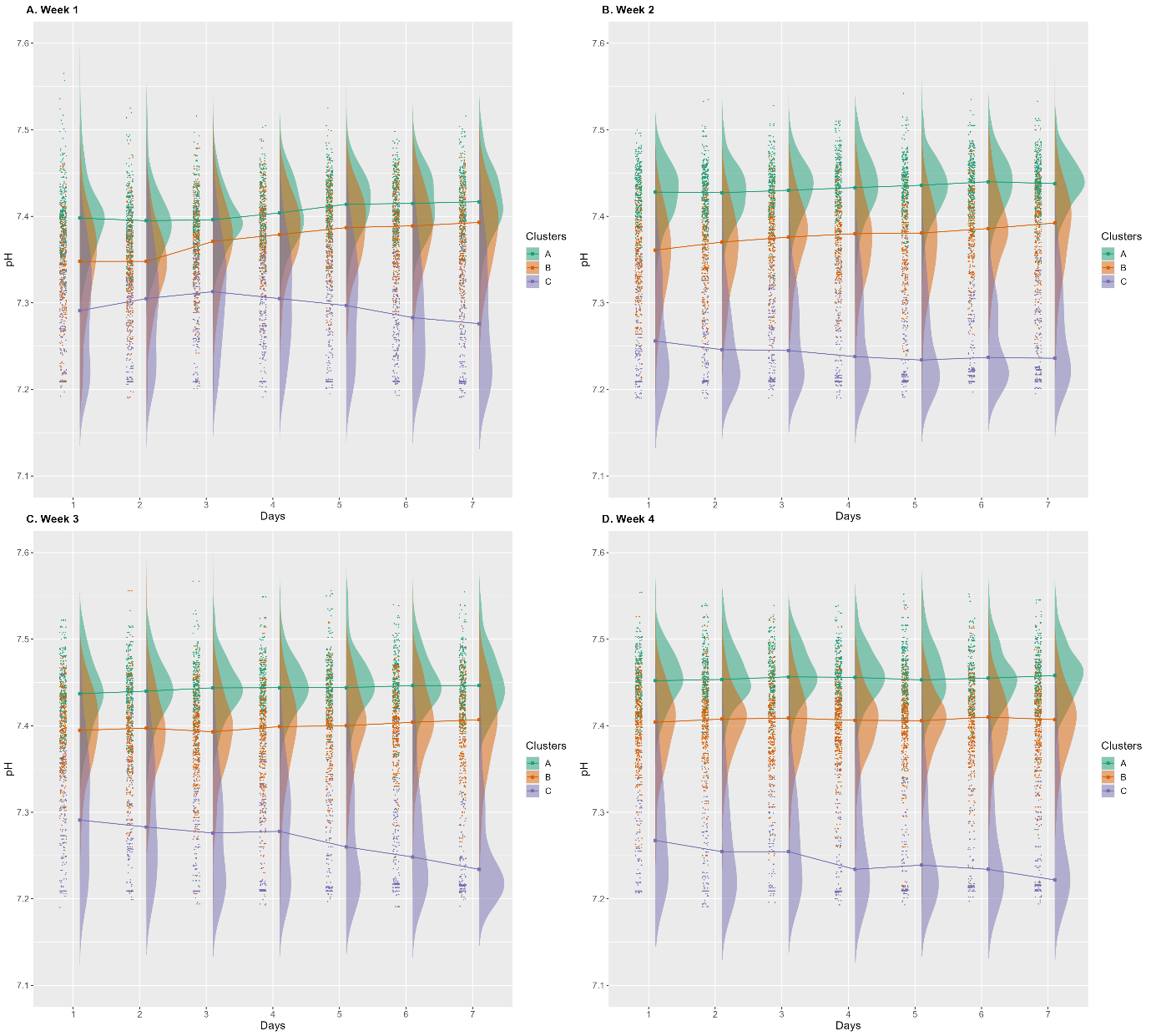


## Fig. S4. For bicarbonate


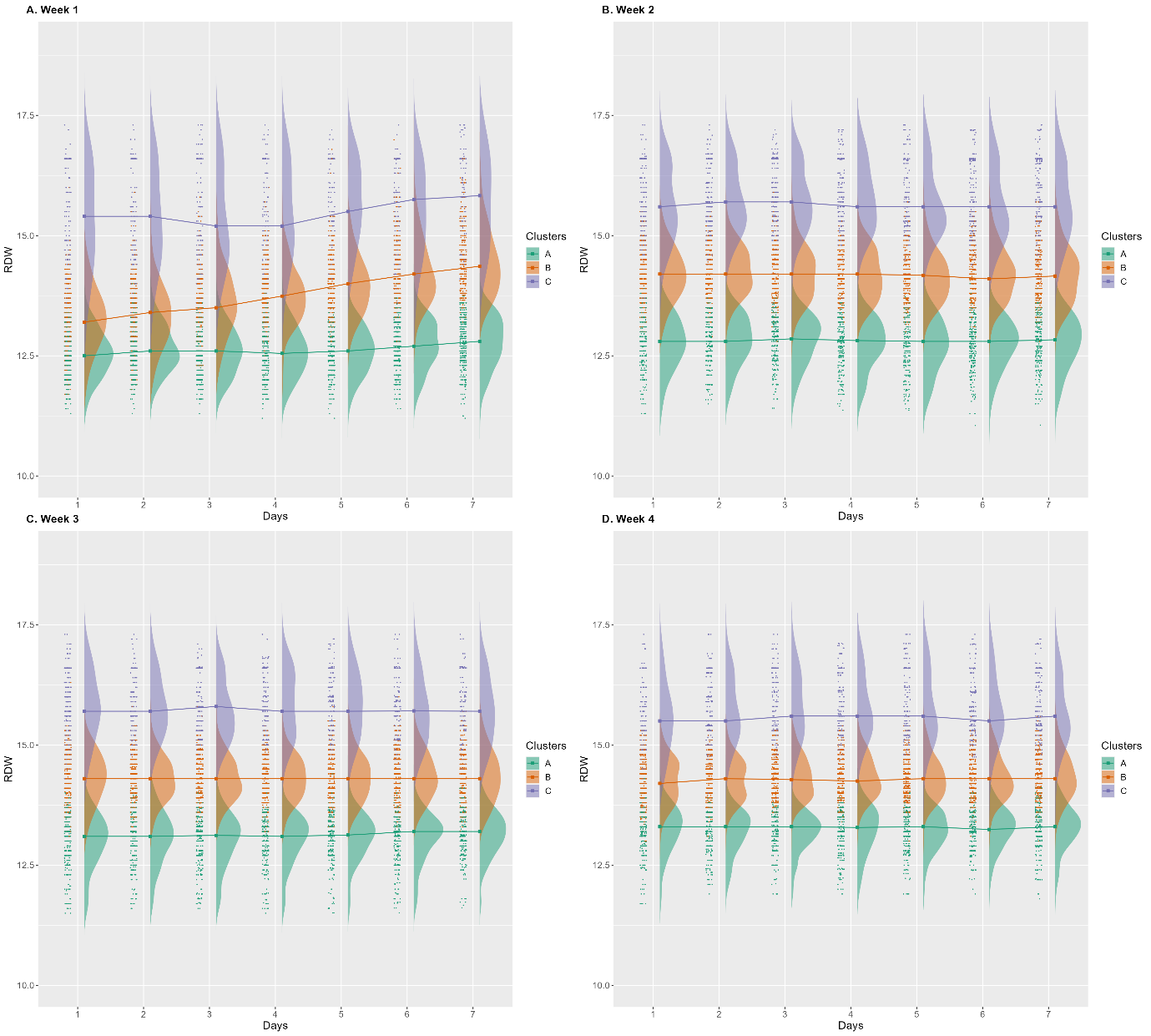


## Fig. S5. For RDW


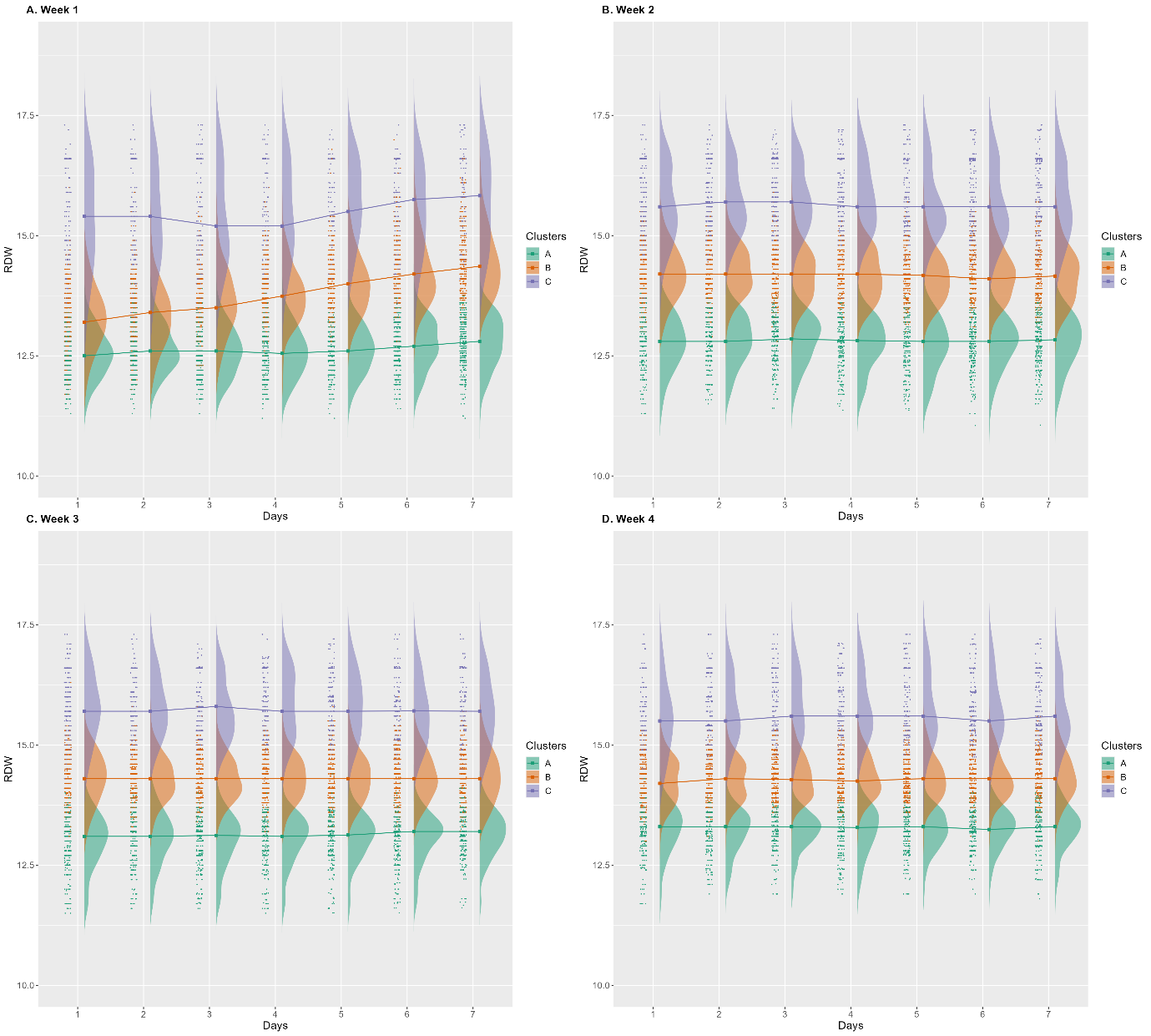


## Fig. S6. For albumin


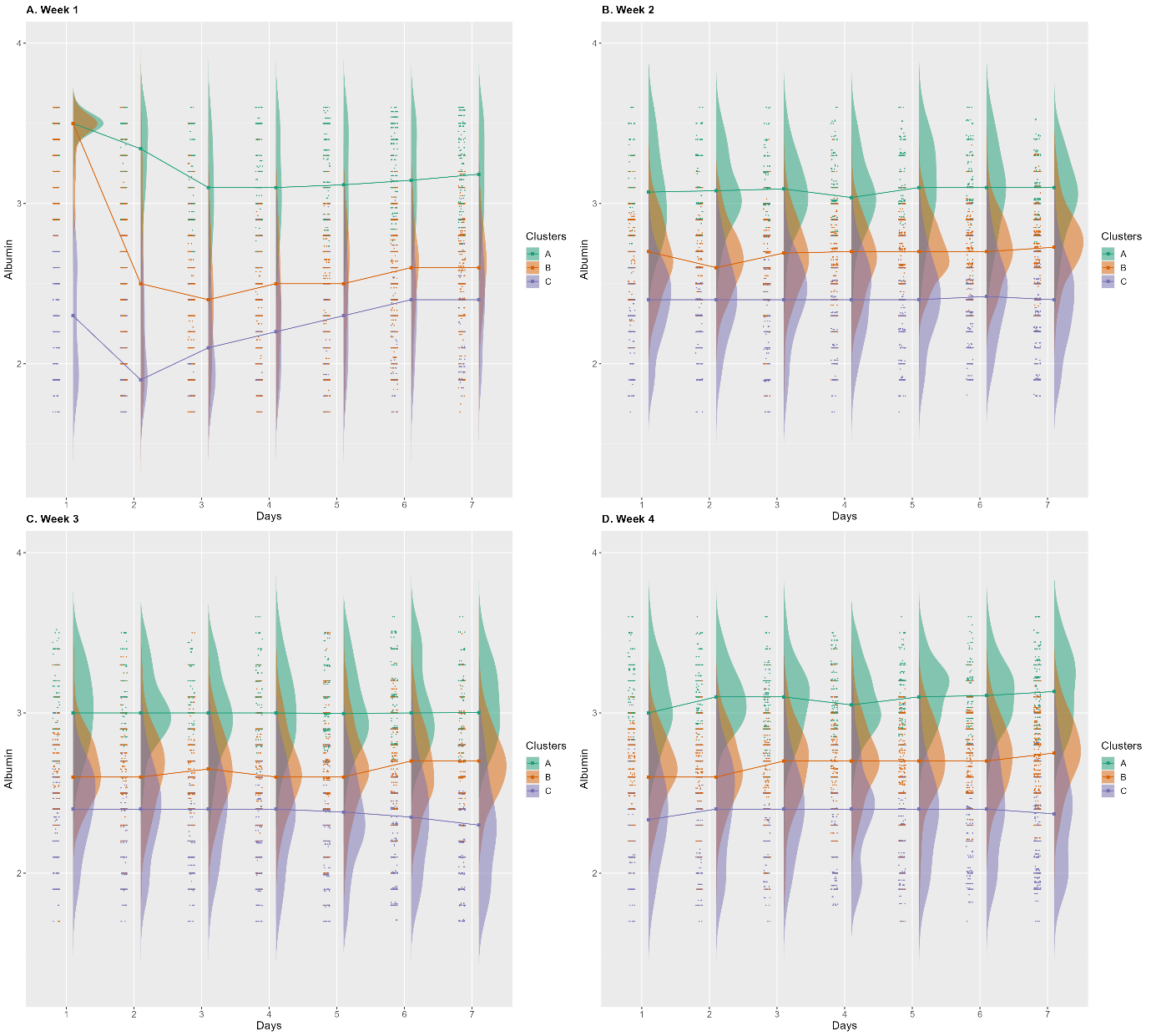


# Figures The kmlShape package Generated Longitudinal Plots for Each Cluster

## Fig. S7. For RDW


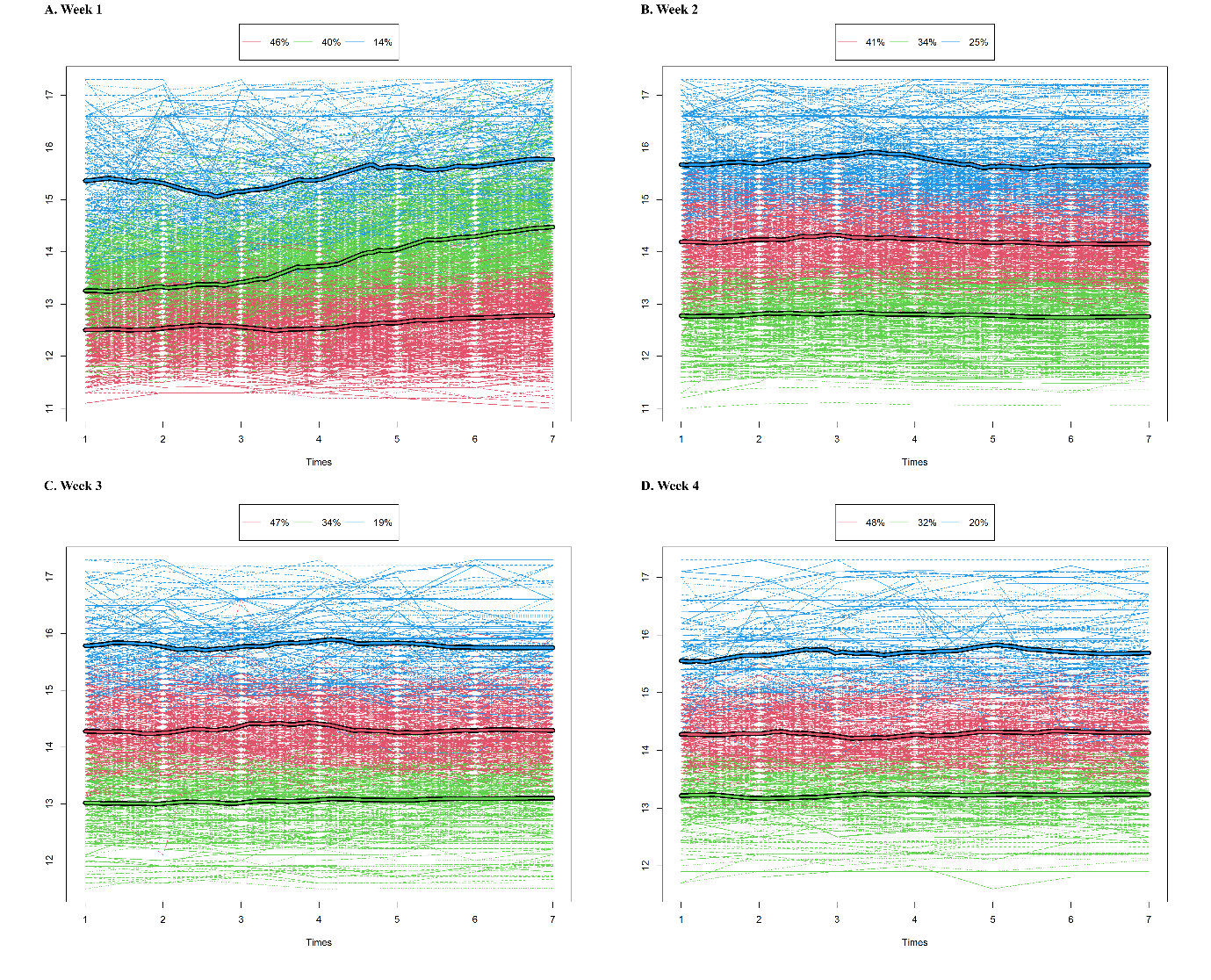


## Fig. S8. For bicarbonate


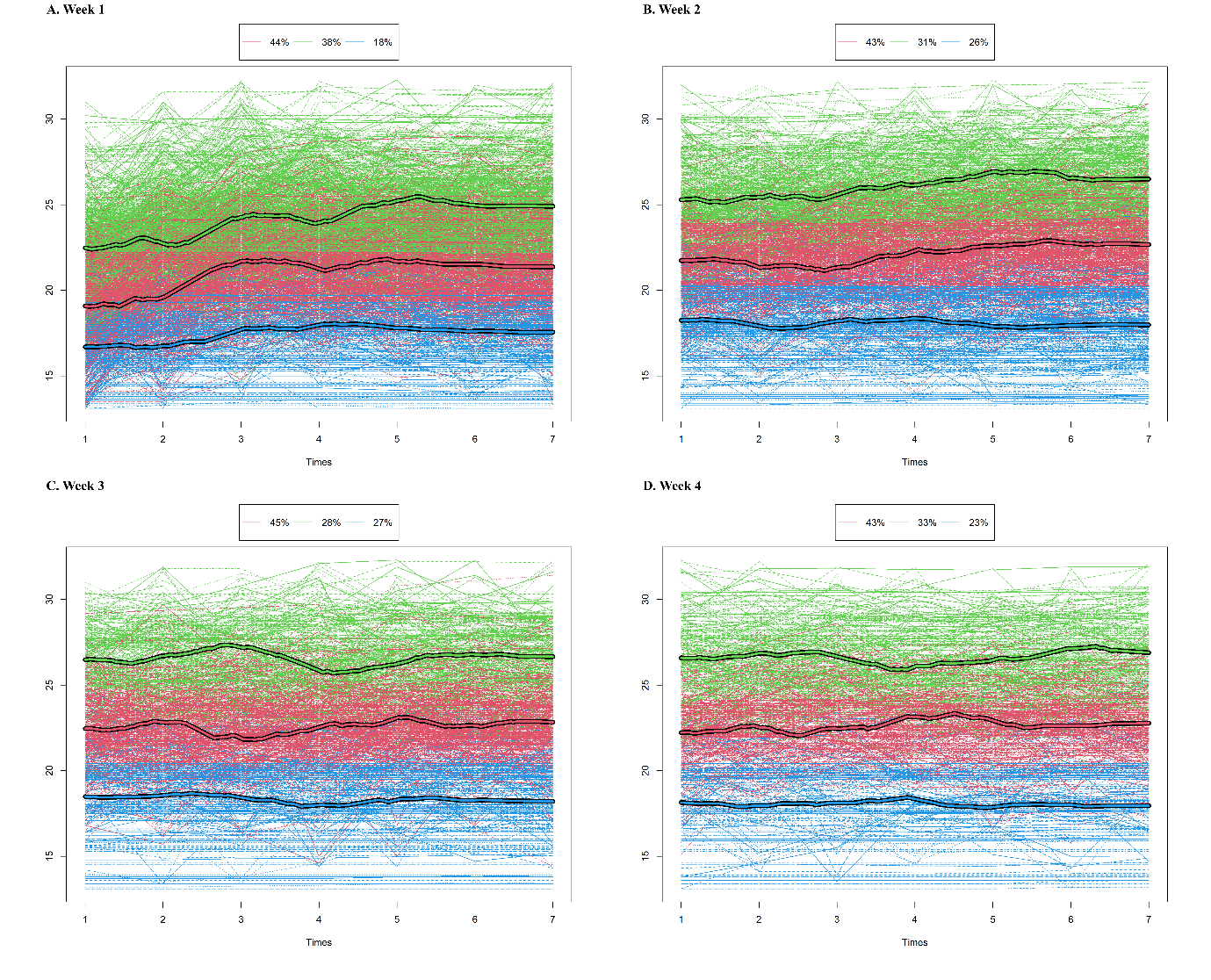


## Fig. S9. For pH


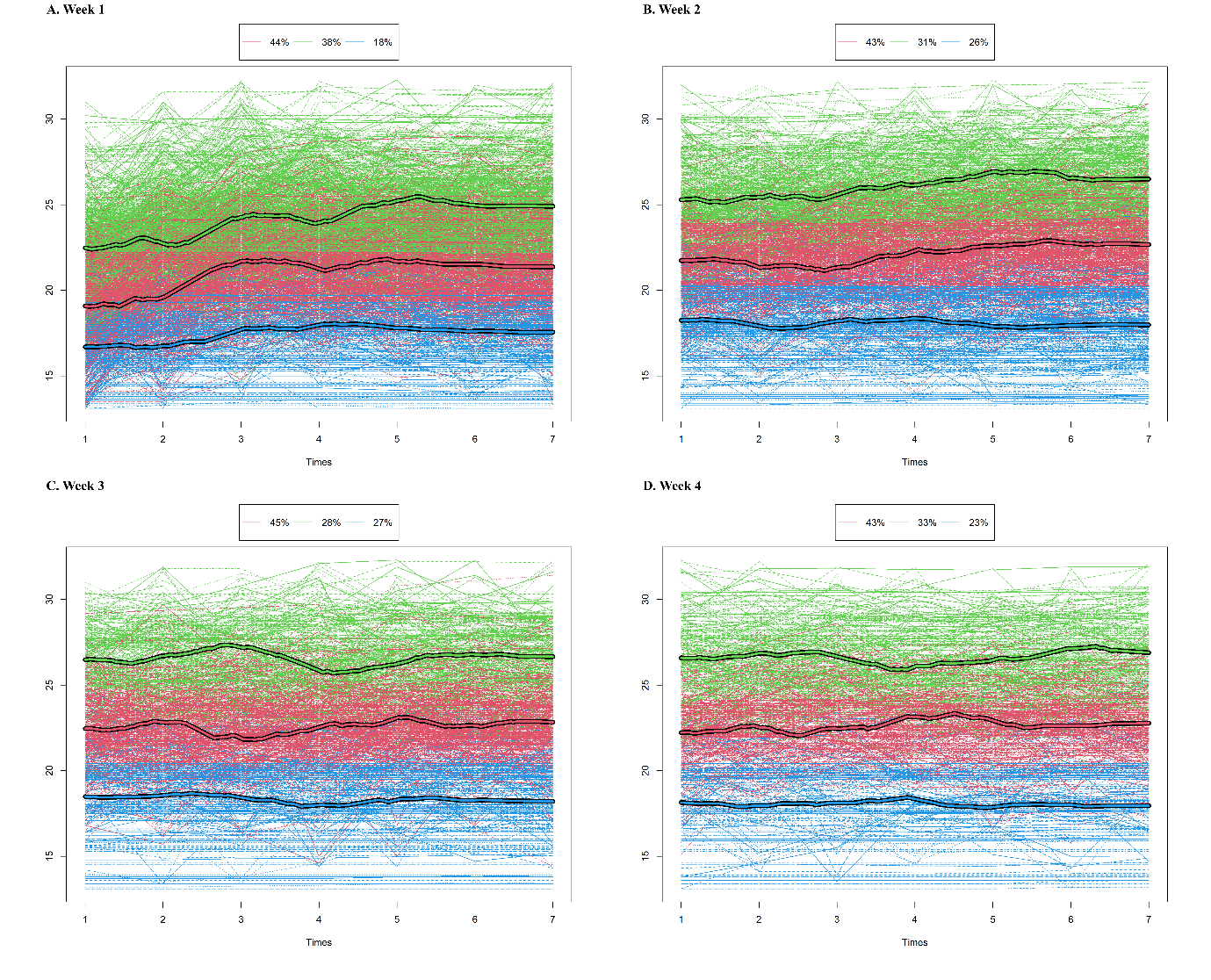


## Fig. S10. For platelet


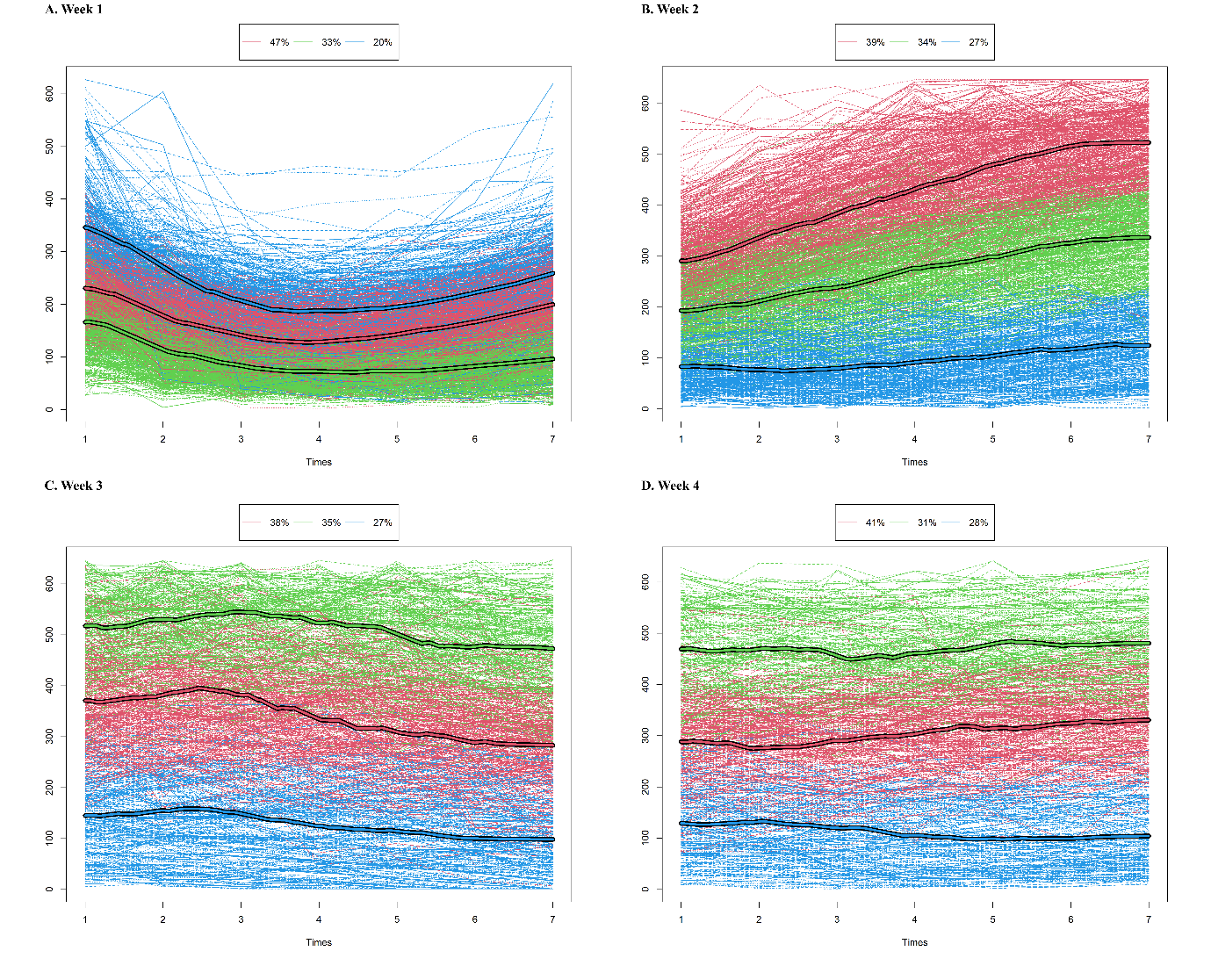


## Fig. S11. For lymphocyte


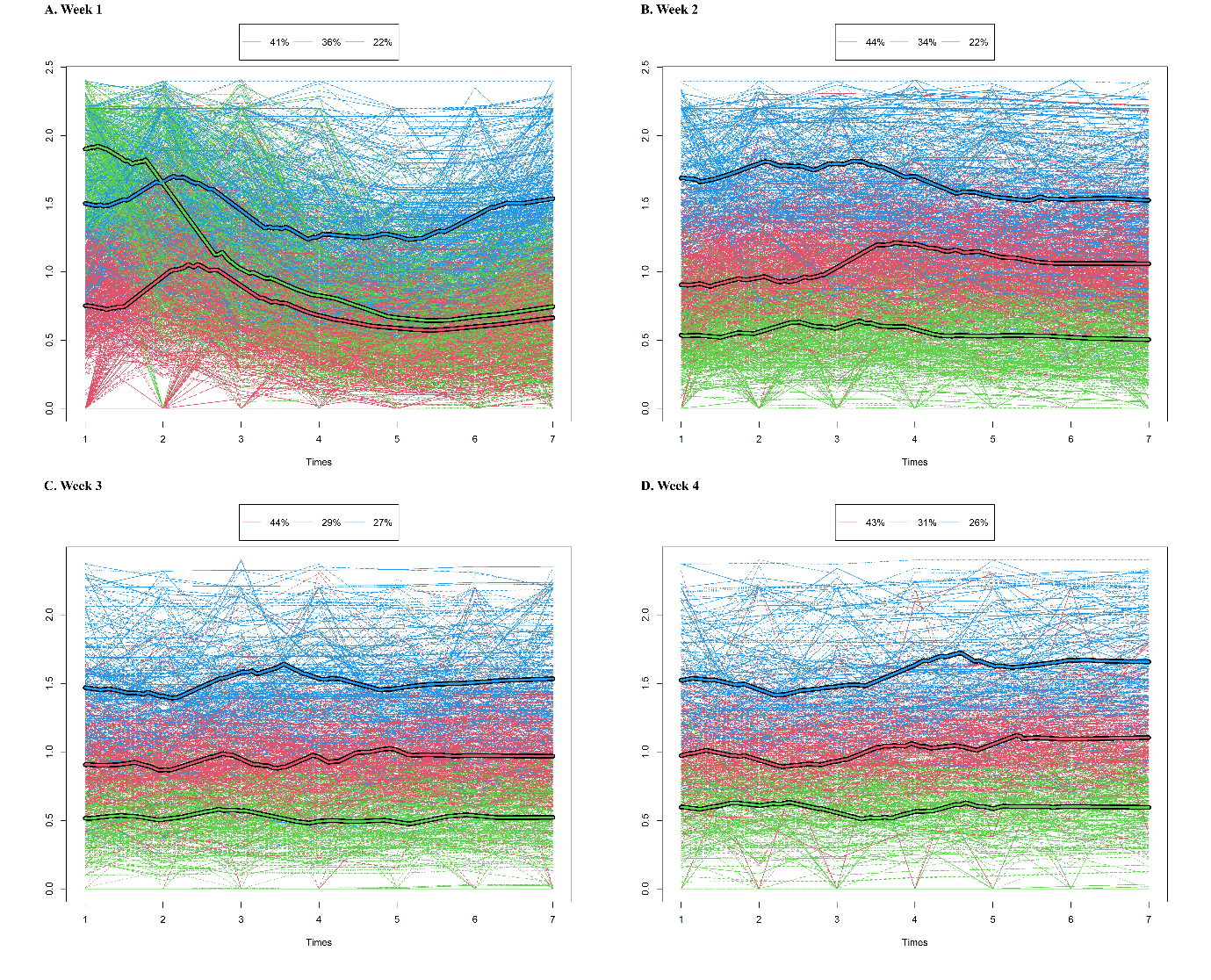


## Fig. S12. For lactate


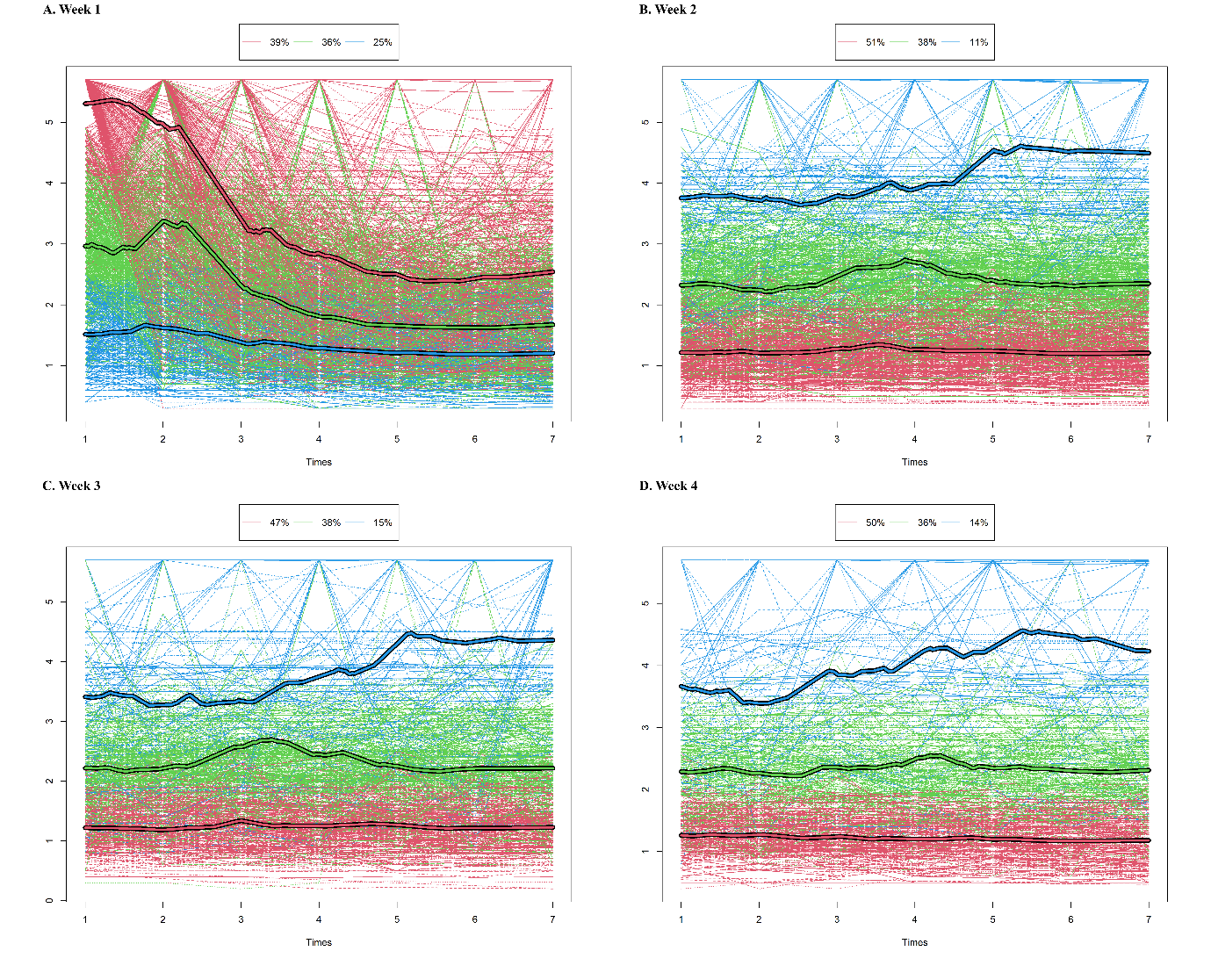


## Fig. S13. For albumin


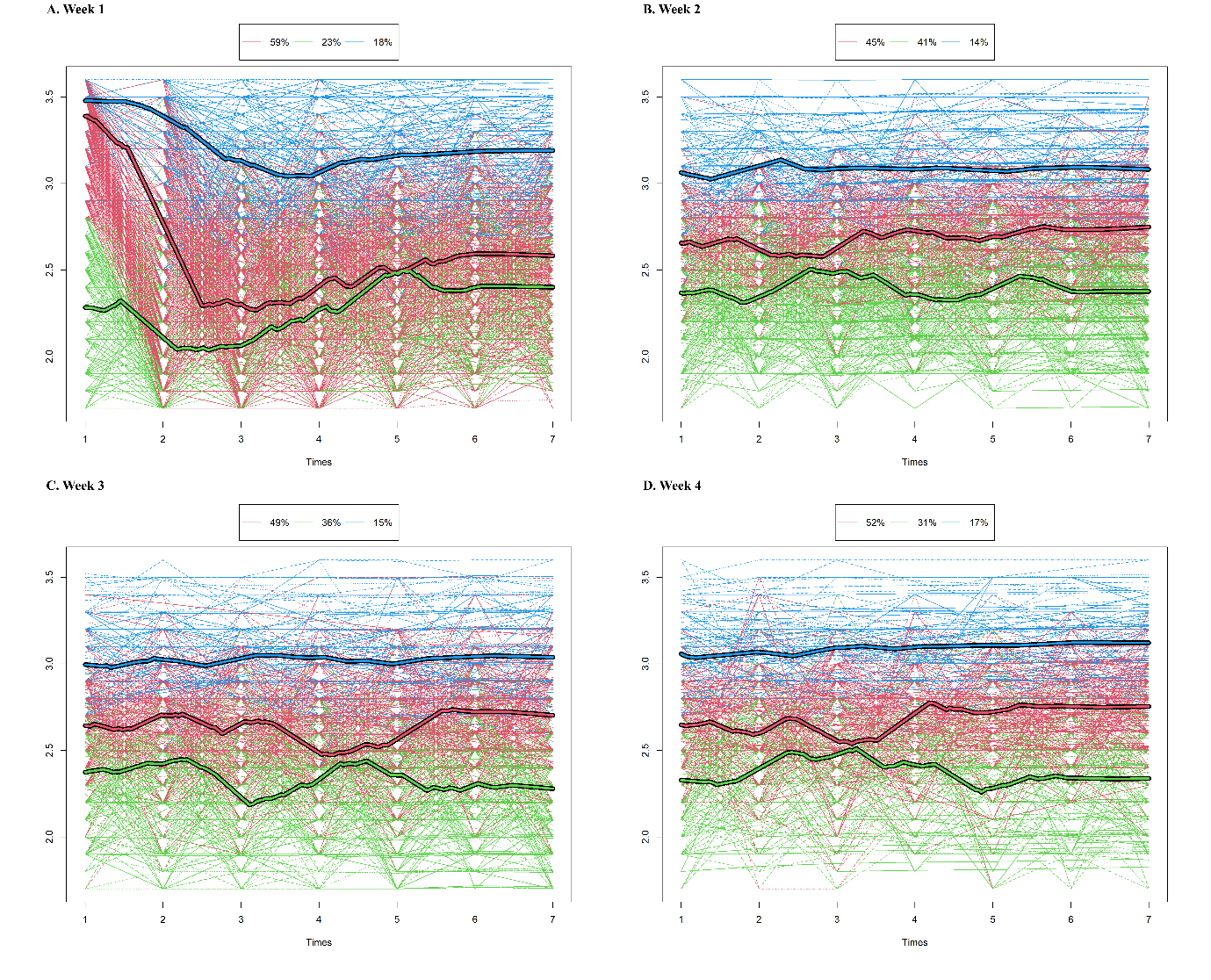


# Tables. The Characteristics and levels Change over time in Positive/Negative Group

## Table S7. For RDW

|  | | **Week 1** | | | | **Week 2** | | | | **Week 3** | | | | **Week 4** | | | |
| --- | --- | --- | --- | --- | --- | --- | --- | --- | --- | --- | --- | --- | --- | --- | --- | --- | --- |
| **Group** | **Variables** | **A**, N = 801 (46.4%) | **B**, N = 690 (40.0%) | **C**, N = 234 (13.6%) | **p-value** | **A**, N = 436 (34.4%) | **B**, N = 516 (40.7%) | **C**, N = 315 (24.9%) | **p-value** | **A**, N = 296 (33.6%) | **B**, N = 416 (47.2%) | **C**, N = 169 (19.2%) | **p-value** | **A**, N = 212 (32.0%) | **B**, N = 319 (48.2%) | **C**, N = 131 (19.8%) | **p-value** |
| Demographics | Mortality | 22 (2.7%) | 230 (33.3%) | 136 (58.1%) | <0.001 | 4 (0.9%) | 117 (22.7%) | 182 (57.8%) | <0.001 | 5 (1.7%) | 88 (21.2%) | 94 (55.6%) | <0.001 | 5 (2.4%) | 47 (14.7%) | 60 (45.8%) | <0.001 |
|  | Patient Age |  |  |  | <0.001 |  |  |  | <0.001 |  |  |  | 0.001 |  |  |  | 0.001 |
|  | Median [IQR] | 48 [38, 57] | 51 [41, 60] | 56 [44, 71] |  | 48 [39, 56] | 52 [42, 62] | 53 [43, 63] |  | 48 [40, 57] | 52 [42, 61] | 53 [42, 63] |  | 50 [41, 58] | 51 [42, 61] | 56 [44, 70] |  |
|  | Sex |  |  |  | <0.001 |  |  |  | <0.001 |  |  |  | 0.004 |  |  |  | 0.001 |
|  | Male | 680 (84.9%) | 548 (79.4%) | 172 (73.5%) |  | 367 (84.2%) | 385 (74.6%) | 260 (82.5%) |  | 252 (85.1%) | 315 (75.7%) | 128 (75.7%) |  | 183 (86.3%) | 236 (74.0%) | 100 (76.3%) |  |
|  | Female | 121 (15.1%) | 142 (20.6%) | 62 (26.5%) |  | 69 (15.8%) | 131 (25.4%) | 55 (17.5%) |  | 44 (14.9%) | 101 (24.3%) | 41 (24.3%) |  | 29 (13.7%) | 83 (26.0%) | 31 (23.7%) |  |
|  | TBSA |  |  |  | <0.001 |  |  |  | <0.001 |  |  |  | <0.001 |  |  |  | <0.001 |
|  | Median [IQR] | 23 [14, 33] | 40 [25, 61] | 40 [20, 69] |  | 25 [16, 35] | 39 [25, 55] | 52 [30, 72] |  | 30 [20, 41] | 40 [27, 60] | 48 [28, 67] |  | 35 [21, 45] | 40 [26, 60] | 39 [27, 60] |  |
|  | Inhalation | 287 (35.8%) | 352 (51.0%) | 124 (53.0%) | <0.001 | 169 (38.8%) | 234 (45.3%) | 171 (54.3%) | <0.001 | 124 (41.9%) | 192 (46.2%) | 80 (47.3%) | 0.431 | 87 (41.0%) | 157 (49.2%) | 65 (49.6%) | 0.135 |
|  | LOICU |  |  |  | <0.001 |  |  |  | <0.001 |  |  |  | <0.001 |  |  |  | <0.001 |
|  | Median [IQR] | 11 [6, 22] | 23 [11, 39] | 15 [8, 32] |  | 17 [11, 27] | 28 [17, 43] | 22 [13, 42] |  | 26 [20, 35] | 35 [25, 52] | 25 [19, 44] |  | 32 [26, 40] | 38 [30, 57] | 35 [26, 48] |  |
| RDW value | Day 1 |  |  |  | <0.001 |  |  |  | <0.001 |  |  |  | <0.001 |  |  |  | <0.001 |
|  | Median [IQR] | 12.50 [12.20, 12.80] | 13.20 [12.80, 13.70] | 15.40 [14.53, 16.58] |  | 12.80 [12.40, 13.20] | 14.20 [13.80, 14.60] | 15.60 [15.20, 16.30] |  | 13.10 [12.69, 13.40] | 14.30 [13.90, 14.60] | 15.70 [15.30, 16.30] |  | 13.30 [12.95, 13.50] | 14.20 [13.90, 14.60] | 15.50 [15.00, 15.95] |  |
|  | Day 2 |  |  |  | <0.001 |  |  |  | <0.001 |  |  |  | <0.001 |  |  |  | <0.001 |
|  | Median [IQR] | 12.60 [12.30, 12.90] | 13.40 [13.00, 13.80] | 15.40 [14.60, 16.60] |  | 12.80 [12.40, 13.20] | 14.20 [13.80, 14.60] | 15.70 [15.20, 16.40] |  | 13.10 [12.70, 13.40] | 14.30 [13.90, 14.60] | 15.70 [15.30, 16.40] |  | 13.30 [13.00, 13.50] | 14.30 [13.91, 14.60] | 15.50 [15.13, 16.10] |  |
|  | Day 3 |  |  |  | <0.001 |  |  |  | <0.001 |  |  |  | <0.001 |  |  |  | <0.001 |
|  | Median [IQR] | 12.60 [12.20, 12.90] | 13.50 [13.10, 14.00] | 15.20 [14.50, 16.28] |  | 12.85 [12.50, 13.20] | 14.20 [13.80, 14.60] | 15.70 [15.26, 16.40] |  | 13.12 [12.70, 13.40] | 14.30 [14.00, 14.60] | 15.80 [15.30, 16.30] |  | 13.30 [13.00, 13.50] | 14.28 [14.00, 14.60] | 15.60 [15.25, 16.17] |  |
|  | Day 4 |  |  |  | <0.001 |  |  |  | <0.001 |  |  |  | <0.001 |  |  |  | <0.001 |
|  | Median [IQR] | 12.55 [12.20, 12.90] | 13.74 [13.30, 14.20] | 15.20 [14.60, 16.20] |  | 12.82 [12.48, 13.20] | 14.20 [13.80, 14.50] | 15.60 [15.20, 16.30] |  | 13.10 [12.70, 13.40] | 14.30 [14.00, 14.60] | 15.70 [15.20, 16.40] |  | 13.28 [12.98, 13.57] | 14.25 [14.00, 14.60] | 15.60 [15.25, 16.20] |  |
|  | Day 5 |  |  |  | <0.001 |  |  |  | <0.001 |  |  |  | <0.001 |  |  |  | <0.001 |
|  | Median [IQR] | 12.60 [12.20, 13.00] | 14.00 [13.50, 14.50] | 15.50 [14.80, 16.40] |  | 12.80 [12.41, 13.20] | 14.17 [13.80, 14.50] | 15.60 [15.20, 16.23] |  | 13.13 [12.71, 13.40] | 14.30 [13.90, 14.60] | 15.70 [15.30, 16.40] |  | 13.30 [12.95, 13.53] | 14.30 [14.00, 14.60] | 15.60 [15.20, 16.17] |  |
|  | Day 6 |  |  |  | <0.001 |  |  |  | <0.001 |  |  |  | <0.001 |  |  |  | <0.001 |
|  | Median [IQR] | 12.70 [12.39, 13.10] | 14.20 [13.80, 14.70] | 15.75 [14.88, 16.56] |  | 12.80 [12.40, 13.18] | 14.10 [13.78, 14.50] | 15.60 [15.10, 16.30] |  | 13.20 [12.80, 13.40] | 14.30 [13.95, 14.70] | 15.71 [15.20, 16.30] |  | 13.24 [12.97, 13.50] | 14.30 [14.00, 14.60] | 15.50 [15.20, 16.10] |  |
|  | Day 7 |  |  |  | <0.001 |  |  |  | <0.001 |  |  |  | <0.001 |  |  |  | <0.001 |
|  | Median [IQR] | 12.80 [12.45, 13.20] | 14.36 [13.90, 14.94] | 15.83 [15.06, 16.60] |  | 12.83 [12.40, 13.19] | 14.16 [13.70, 14.50] | 15.60 [15.10, 16.40] |  | 13.20 [12.80, 13.50] | 14.30 [13.90, 14.70] | 15.70 [15.30, 16.30] |  | 13.30 [13.00, 13.54] | 14.30 [14.00, 14.60] | 15.60 [15.20, 16.20] |  |
| Mean of overall median value | RDW |  |  |  | <0.001 |  |  |  | <0.001 |  |  |  | <0.001 |  |  |  | <0.001 |
|  | Median [IQR] | 12.60 [12.30, 13.00] | 13.80 [13.30, 14.30] | 15.50 [14.70, 16.50] |  | 12.80 [12.41, 13.20] | 14.20 [13.80, 14.55] | 15.60 [15.20, 16.34] |  | 13.10 [12.71, 13.40] | 14.30 [13.90, 14.60] | 15.70 [15.27, 16.32] |  | 13.30 [13.00, 13.51] | 14.30 [14.00, 14.60] | 15.60 [15.20, 16.12] |  |

## Table S8. For bicarbonate

|  | | **Week 1** | | | | **Week 2** | | | | **Week 3** | | | | **Week 4** | | | |
| --- | --- | --- | --- | --- | --- | --- | --- | --- | --- | --- | --- | --- | --- | --- | --- | --- | --- |
| **Group** | **Variables** | **A**, N = 640 (37.9%) | **B**, N = 743 (44.0%) | **C**, N = 307 (18.2%) | **p-value** | **A**, N = 388 (31.1%) | **B**, N = 535 (42.9%) | **C**, N = 324 (26.0%) | **p-value** | **A**, N = 242 (27.8%) | **B**, N = 394 (45.3%) | **C**, N = 234 (26.9%) | **p-value** | **A**, N = 219 (33.5%) | **B**, N = 283 (43.3%) | **C**, N = 152 (23.2%) | **p-value** |
| Demographics | Mortality | 48 (7.5%) | 192 (25.8%) | 142 (46.3%) | <0.001 | 34 (8.8%) | 121 (22.6%) | 143 (44.1%) | <0.001 | 18 (7.4%) | 69 (17.5%) | 98 (41.9%) | <0.001 | 9 (4.1%) | 43 (15.2%) | 59 (38.8%) | <0.001 |
|  | Patient Age |  |  |  | <0.001 |  |  |  | <0.001 |  |  |  | <0.001 |  |  |  | 0.037 |
|  | Median [IQR] | 48 [39, 57] | 50 [40, 60] | 55 [44, 64] |  | 48 [39, 57] | 50 [41, 60] | 54 [44, 65] |  | 49 [41, 58] | 50 [40, 59] | 54 [44, 65] |  | 49 [41, 58] | 52 [41, 62] | 53 [44, 64] |  |
|  | Sex |  |  |  | 0.030 |  |  |  | 0.972 |  |  |  | 0.917 |  |  |  | 0.924 |
|  | Male | 540 (84.4%) | 593 (79.8%) | 241 (78.5%) |  | 309 (79.6%) | 429 (80.2%) | 260 (80.2%) |  | 189 (78.1%) | 313 (79.4%) | 185 (79.1%) |  | 170 (77.6%) | 224 (79.2%) | 119 (78.3%) |  |
|  | Female | 100 (15.6%) | 150 (20.2%) | 66 (21.5%) |  | 79 (20.4%) | 106 (19.8%) | 64 (19.8%) |  | 53 (21.9%) | 81 (20.6%) | 49 (20.9%) |  | 49 (22.4%) | 59 (20.8%) | 33 (21.7%) |  |
|  | TBSA |  |  |  | <0.001 |  |  |  | <0.001 |  |  |  | <0.001 |  |  |  | <0.001 |
|  | Median [IQR] | 24 [14, 34] | 35 [21, 60] | 40 [24, 61] |  | 30 [20, 42] | 36 [23, 55] | 40 [25, 61] |  | 30 [20, 42] | 40 [26, 56] | 40 [26, 60] |  | 35 [21, 46] | 40 [24, 59] | 42 [30, 60] |  |
|  | Inhalation | 273 (42.7%) | 370 (49.8%) | 115 (37.5%) | <0.001 | 197 (50.8%) | 263 (49.2%) | 109 (33.6%) | <0.001 | 121 (50.0%) | 194 (49.2%) | 78 (33.3%) | <0.001 | 113 (51.6%) | 137 (48.4%) | 57 (37.5%) | 0.022 |
|  | LOICU |  |  |  | <0.001 |  |  |  | 0.005 |  |  |  | 0.001 |  |  |  | 0.004 |
|  | Median [IQR] | 12 [6, 24] | 20 [9, 35] | 17 [8, 33] |  | 21 [12, 34] | 25 [14, 38] | 22 [13, 36] |  | 28 [21, 41] | 32 [24, 43] | 26 [20, 44] |  | 34 [27, 44] | 37 [29, 56] | 35 [26, 48] |  |
| Bicarbonate value | Day 1 |  |  |  | <0.001 |  |  |  | <0.001 |  |  |  | <0.001 |  |  |  | <0.001 |
|  | Median [IQR] | 22.5 [21.0, 23.9] | 19.2 [17.2, 20.9] | 16.5 [15.6, 18.2] |  | 25.2 [23.9, 27.0] | 21.7 [20.5, 23.0] | 18.2 [16.6, 19.7] |  | 26.5 [25.0, 27.9] | 22.2 [21.2, 23.9] | 18.5 [16.9, 19.9] |  | 26.7 [25.0, 28.3] | 22.3 [21.0, 23.5] | 18.3 [16.6, 19.6] |  |
|  | Day 2 |  |  |  | <0.001 |  |  |  | <0.001 |  |  |  | <0.001 |  |  |  | <0.001 |
|  | Median [IQR] | 23.6 [22.4, 25.1] | 20.8 [19.2, 22.2] | 17.4 [16.2, 18.6] |  | 25.4 [24.0, 27.1] | 22.1 [20.6, 23.2] | 18.1 [16.4, 19.6] |  | 26.6 [24.9, 28.2] | 22.5 [21.1, 23.8] | 18.5 [17.0, 19.7] |  | 26.7 [25.0, 28.4] | 22.6 [21.3, 23.9] | 18.1 [16.4, 19.6] |  |
|  | Day 3 |  |  |  | <0.001 |  |  |  | <0.001 |  |  |  | <0.001 |  |  |  | <0.001 |
|  | Median [IQR] | 24.2 [22.8, 25.9] | 21.5 [20.2, 23.1] | 17.8 [16.4, 19.0] |  | 25.6 [24.3, 27.3] | 22.1 [20.8, 23.3] | 18.1 [16.5, 19.5] |  | 26.4 [24.9, 28.4] | 22.5 [21.1, 23.9] | 18.5 [16.6, 19.8] |  | 26.8 [25.1, 28.5] | 22.6 [21.3, 24.0] | 18.0 [16.4, 19.5] |  |
|  | Day 4 |  |  |  | <0.001 |  |  |  | <0.001 |  |  |  | <0.001 |  |  |  | <0.001 |
|  | Median [IQR] | 24.3 [23.0, 26.3] | 21.4 [20.1, 22.9] | 17.9 [16.4, 18.9] |  | 26.0 [24.6, 27.7] | 22.1 [20.9, 23.4] | 18.1 [16.4, 19.4] |  | 26.5 [24.9, 28.4] | 22.6 [21.2, 23.9] | 18.5 [16.7, 19.7] |  | 26.5 [25.0, 28.4] | 22.6 [21.5, 23.9] | 18.0 [16.4, 19.6] |  |
|  | Day 5 |  |  |  | <0.001 |  |  |  | <0.001 |  |  |  | <0.001 |  |  |  | <0.001 |
|  | Median [IQR] | 24.2 [22.8, 26.2] | 21.4 [20.0, 22.8] | 17.9 [16.4, 18.9] |  | 26.2 [24.9, 27.7] | 22.4 [21.1, 23.7] | 18.1 [16.5, 19.6] |  | 26.7 [25.2, 28.6] | 22.6 [21.4, 24.2] | 18.5 [16.6, 19.9] |  | 26.5 [24.8, 28.5] | 22.6 [21.3, 23.8] | 18.0 [16.4, 19.5] |  |
|  | Day 6 |  |  |  | <0.001 |  |  |  | <0.001 |  |  |  | <0.001 |  |  |  | <0.001 |
|  | Median [IQR] | 24.4 [23.0, 26.3] | 21.3 [19.9, 22.9] | 17.7 [16.4, 18.9] |  | 26.2 [25.0, 27.8] | 22.4 [21.2, 23.8] | 18.2 [16.5, 19.5] |  | 26.6 [25.1, 28.5] | 22.7 [21.4, 24.2] | 18.5 [16.7, 19.9] |  | 26.8 [25.2, 28.5] | 22.8 [21.5, 23.9] | 18.2 [16.4, 19.7] |  |
|  | Day 7 |  |  |  | <0.001 |  |  |  | <0.001 |  |  |  | <0.001 |  |  |  | <0.001 |
|  | Median [IQR] | 24.6 [23.1, 26.5] | 21.3 [20.0, 22.8] | 17.7 [16.4, 18.8] |  | 26.5 [25.0, 28.0] | 22.5 [21.3, 23.9] | 18.0 [16.4, 19.6] |  | 26.9 [25.1, 28.4] | 22.6 [21.4, 24.1] | 18.3 [16.7, 19.7] |  | 27.0 [25.1, 28.8] | 22.6 [21.4, 23.9] | 18.0 [16.4, 19.6] |  |
| Mean of overall median value | Bicarbonate |  |  |  | <0.001 |  |  |  | <0.001 |  |  |  | <0.001 |  |  |  | <0.001 |
|  | Median [IQR] | 23.9 [22.5, 25.8] | 21.1 [19.6, 22.5] | 17.6 [16.4, 18.8] |  | 25.9 [24.5, 27.6] | 22.2 [20.9, 23.5] | 18.1 [16.4, 19.5] |  | 26.6 [25.0, 28.3] | 22.5 [21.3, 24.0] | 18.5 [16.7, 19.8] |  | 26.7 [25.0, 28.5] | 22.6 [21.3, 23.9] | 18.1 [16.4, 19.6] |  |

## Table S9. For pH

|  | | **Week 1** | | | | **Week 2** | | | | **Week 3** | | | | **Week 4** | | | |
| --- | --- | --- | --- | --- | --- | --- | --- | --- | --- | --- | --- | --- | --- | --- | --- | --- | --- |
| **Group** | **Variables** | **A**, N = 562 (32.6%) | **B**, N = 673 (39.1%) | **C**, N = 487 (28.3%) | **p-value** | **A**, N = 526 (41.6%) | **B**, N = 466 (36.8%) | **C**, N = 273 (21.6%) | **p-value** | **A**, N = 292 (33.1%) | **B**, N = 403 (45.7%) | **C**, N = 187 (21.2%) | **p-value** | **A**, N = 218 (32.9%) | **B**, N = 344 (52.0%) | **C**, N = 100 (15.1%) | **p-value** |
| Demographics | Mortality | 25 (4.4%) | 70 (10.4%) | 293 (60.2%) | <0.001 | 12 (2.3%) | 76 (16.3%) | 216 (79.1%) | <0.001 | 13 (4.5%) | 40 (9.9%) | 135 (72.2%) | <0.001 | 7 (3.2%) | 32 (9.3%) | 73 (73.0%) | <0.001 |
|  | Patient Age |  |  |  | <0.001 |  |  |  | 0.007 |  |  |  | <0.001 |  |  |  | 0.229 |
|  | Median [IQR] | 50 [41, 61] | 49 [38, 58] | 52 [42, 61] |  | 50 [41, 60] | 50 [40, 59] | 53 [43, 65] |  | 50 [42, 62] | 49 [40, 58] | 54 [45, 66] |  | 52 [40, 62] | 51 [42, 61] | 54 [45, 64] |  |
|  | Sex |  |  |  | <0.001 |  |  |  | <0.001 |  |  |  | 0.050 |  |  |  | 0.612 |
|  | Male | 435 (77.4%) | 547 (81.3%) | 418 (85.8%) |  | 395 (75.1%) | 385 (82.6%) | 232 (85.0%) |  | 217 (74.3%) | 330 (81.9%) | 149 (79.7%) |  | 166 (76.1%) | 273 (79.4%) | 80 (80.0%) |  |
|  | Female | 127 (22.6%) | 126 (18.7%) | 69 (14.2%) |  | 131 (24.9%) | 81 (17.4%) | 41 (15.0%) |  | 75 (25.7%) | 73 (18.1%) | 38 (20.3%) |  | 52 (23.9%) | 71 (20.6%) | 20 (20.0%) |  |
|  | TBSA |  |  |  | <0.001 |  |  |  | <0.001 |  |  |  | <0.001 |  |  |  | <0.001 |
|  | Median [IQR] | 22 [12, 31] | 29 [18, 42] | 60 [35, 77] |  | 28 [19, 38] | 35 [22, 53] | 61 [40, 80] |  | 30 [20, 41] | 38 [24, 51] | 60 [38, 75] |  | 35 [21, 45] | 38 [24, 51] | 56 [36, 70] |  |
|  | Inhalation | 167 (29.7%) | 282 (41.9%) | 315 (64.7%) | <0.001 | 197 (37.5%) | 208 (44.6%) | 170 (62.3%) | <0.001 | 115 (39.4%) | 184 (45.7%) | 98 (52.4%) | 0.018 | 86 (39.4%) | 163 (47.4%) | 60 (60.0%) | 0.004 |
|  | LOICU |  |  |  | <0.001 |  |  |  | <0.001 |  |  |  | <0.001 |  |  |  | <0.001 |
|  | Median [IQR] | 10 [5, 22] | 18 [9, 33] | 19 [10, 35] |  | 20 [11, 30] | 30 [19, 45] | 18 [12, 30] |  | 27 [21, 39] | 34 [25, 45] | 25 [19, 39] |  | 34 [27, 46] | 38 [30, 53] | 29 [25, 47] |  |
| pH value | Day 1 |  |  |  | <0.001 |  |  |  | <0.001 |  |  |  | <0.001 |  |  |  | <0.001 |
|  | Median [IQR] | 7.40 [7.37, 7.42] | 7.35 [7.31, 7.38] | 7.29 [7.22, 7.34] |  | 7.43 [7.40, 7.45] | 7.36 [7.33, 7.39] | 7.26 [7.21, 7.31] |  | 7.44 [7.41, 7.46] | 7.39 [7.36, 7.42] | 7.29 [7.22, 7.35] |  | 7.45 [7.43, 7.48] | 7.40 [7.37, 7.43] | 7.27 [7.22, 7.32] |  |
|  | Day 2 |  |  |  | <0.001 |  |  |  | <0.001 |  |  |  | <0.001 |  |  |  | <0.001 |
|  | Median [IQR] | 7.39 [7.37, 7.42] | 7.35 [7.32, 7.38] | 7.30 [7.25, 7.34] |  | 7.43 [7.41, 7.45] | 7.37 [7.34, 7.40] | 7.25 [7.21, 7.30] |  | 7.44 [7.42, 7.46] | 7.40 [7.36, 7.42] | 7.28 [7.23, 7.35] |  | 7.45 [7.44, 7.48] | 7.41 [7.38, 7.43] | 7.25 [7.21, 7.31] |  |
|  | Day 3 |  |  |  | <0.001 |  |  |  | <0.001 |  |  |  | <0.001 |  |  |  | <0.001 |
|  | Median [IQR] | 7.40 [7.38, 7.42] | 7.37 [7.34, 7.40] | 7.31 [7.27, 7.35] |  | 7.43 [7.41, 7.45] | 7.38 [7.34, 7.40] | 7.24 [7.21, 7.30] |  | 7.44 [7.42, 7.47] | 7.39 [7.36, 7.42] | 7.28 [7.21, 7.33] |  | 7.46 [7.44, 7.48] | 7.41 [7.38, 7.43] | 7.25 [7.21, 7.31] |  |
|  | Day 4 |  |  |  | <0.001 |  |  |  | <0.001 |  |  |  | <0.001 |  |  |  | <0.001 |
|  | Median [IQR] | 7.40 [7.38, 7.43] | 7.38 [7.35, 7.41] | 7.30 [7.26, 7.35] |  | 7.43 [7.41, 7.45] | 7.38 [7.35, 7.40] | 7.24 [7.21, 7.29] |  | 7.44 [7.42, 7.47] | 7.40 [7.36, 7.43] | 7.28 [7.21, 7.32] |  | 7.46 [7.44, 7.48] | 7.41 [7.38, 7.42] | 7.23 [7.21, 7.30] |  |
|  | Day 5 |  |  |  | <0.001 |  |  |  | <0.001 |  |  |  | <0.001 |  |  |  | <0.001 |
|  | Median [IQR] | 7.41 [7.39, 7.44] | 7.39 [7.36, 7.41] | 7.30 [7.24, 7.35] |  | 7.44 [7.41, 7.45] | 7.38 [7.34, 7.41] | 7.23 [7.21, 7.30] |  | 7.44 [7.43, 7.47] | 7.40 [7.37, 7.43] | 7.26 [7.21, 7.32] |  | 7.45 [7.43, 7.48] | 7.41 [7.38, 7.43] | 7.24 [7.21, 7.30] |  |
|  | Day 6 |  |  |  | <0.001 |  |  |  | <0.001 |  |  |  | <0.001 |  |  |  | <0.001 |
|  | Median [IQR] | 7.42 [7.39, 7.44] | 7.39 [7.36, 7.42] | 7.28 [7.23, 7.33] |  | 7.44 [7.42, 7.46] | 7.39 [7.35, 7.41] | 7.24 [7.21, 7.30] |  | 7.45 [7.43, 7.47] | 7.40 [7.38, 7.43] | 7.25 [7.22, 7.31] |  | 7.46 [7.44, 7.47] | 7.41 [7.38, 7.43] | 7.23 [7.21, 7.32] |  |
|  | Day 7 |  |  |  | <0.001 |  |  |  | <0.001 |  |  |  | <0.001 |  |  |  | <0.001 |
|  | Median [IQR] | 7.42 [7.39, 7.45] | 7.39 [7.36, 7.42] | 7.28 [7.21, 7.33] |  | 7.44 [7.42, 7.46] | 7.39 [7.35, 7.42] | 7.24 [7.22, 7.30] |  | 7.45 [7.43, 7.47] | 7.41 [7.37, 7.43] | 7.23 [7.21, 7.30] |  | 7.46 [7.44, 7.48] | 7.41 [7.38, 7.43] | 7.22 [7.21, 7.32] |  |
| Mean of overall median value | pH |  |  |  | <0.001 |  |  |  | <0.001 |  |  |  | <0.001 |  |  |  | <0.001 |
|  | Median [IQR] | 7.40 [7.38, 7.43] | 7.37 [7.34, 7.40] | 7.30 [7.24, 7.34] |  | 7.43 [7.41, 7.45] | 7.38 [7.34, 7.41] | 7.24 [7.21, 7.30] |  | 7.44 [7.42, 7.47] | 7.40 [7.37, 7.43] | 7.27 [7.21, 7.33] |  | 7.46 [7.44, 7.48] | 7.41 [7.38, 7.43] | 7.24 [7.21, 7.31] |  |

## Table S10. For platelet

|  | | **Week 1** | | | | **Week 2** | | | | **Week 3** | | | | **Week 4** | | | |
| --- | --- | --- | --- | --- | --- | --- | --- | --- | --- | --- | --- | --- | --- | --- | --- | --- | --- |
| **Group** | **Variables** | **A**, N = 807 (46.8%) | **B**, N = 344 (19.9%) | **C**, N = 574 (33.3%) | **p-value** | **A**, N = 493 (38.9%) | **B**, N = 433 (34.2%) | **C**, N = 341 (26.9%) | **p-value** | **A**, N = 310 (35.2%) | **B**, N = 333 (37.8%) | **C**, N = 238 (27.0%) | **p-value** | **A**, N = 206 (31.1%) | **B**, N = 273 (41.2%) | **C**, N = 183 (27.6%) | **p-value** |
| Demographics | Mortality | 77 (9.5%) | 37 (10.8%) | 274 (47.7%) | <0.001 | 12 (2.4%) | 56 (12.9%) | 235 (68.9%) | <0.001 | 5 (1.6%) | 44 (13.2%) | 138 (58.0%) | <0.001 | 4 (1.9%) | 15 (5.5%) | 93 (50.8%) | <0.001 |
|  | Patient Age |  |  |  | <0.001 |  |  |  | <0.001 |  |  |  | <0.001 |  |  |  | <0.001 |
|  | Median [IQR] | 49 [40, 58] | 48 [37, 59] | 53 [43, 63] |  | 47 [38, 55] | 53 [44, 63] | 55 [43, 65] |  | 47 [39, 56] | 52 [43, 61] | 54 [44, 67] |  | 48 [40, 57] | 51 [42, 60] | 56 [45, 70] |  |
|  | Sex |  |  |  | <0.001 |  |  |  | 0.294 |  |  |  | 0.052 |  |  |  | 0.317 |
|  | Male | 669 (82.9%) | 249 (72.4%) | 482 (84.0%) |  | 396 (80.3%) | 336 (77.6%) | 280 (82.1%) |  | 258 (83.2%) | 253 (76.0%) | 184 (77.3%) |  | 169 (82.0%) | 209 (76.6%) | 141 (77.0%) |  |
|  | Female | 138 (17.1%) | 95 (27.6%) | 92 (16.0%) |  | 97 (19.7%) | 97 (22.4%) | 61 (17.9%) |  | 52 (16.8%) | 80 (24.0%) | 54 (22.7%) |  | 37 (18.0%) | 64 (23.4%) | 42 (23.0%) |  |
|  | TBSA |  |  |  | <0.001 |  |  |  | <0.001 |  |  |  | <0.001 |  |  |  | 0.016 |
|  | Median [IQR] | 26 [16, 40] | 24 [14, 35] | 46 [26, 70] |  | 30 [20, 40] | 31 [20, 47] | 60 [34, 77] |  | 33 [21, 45] | 37 [23, 51] | 50 [29, 70] |  | 38 [24, 48] | 37 [24, 51] | 41 [25, 61] |  |
|  | Inhalation | 315 (39.0%) | 148 (43.0%) | 300 (52.3%) | <0.001 | 223 (45.2%) | 167 (38.6%) | 184 (54.0%) | <0.001 | 149 (48.1%) | 141 (42.3%) | 106 (44.5%) | 0.350 | 111 (53.9%) | 115 (42.1%) | 83 (45.4%) | 0.033 |
|  | LOICU |  |  |  | <0.001 |  |  |  | 0.315 |  |  |  | <0.001 |  |  |  | 0.007 |
|  | Median [IQR] | 14 [6, 28] | 12 [7, 26] | 20 [10, 36] |  | 22 [13, 34] | 25 [13, 38] | 20 [13, 38] |  | 27 [21, 37] | 33 [25, 46] | 28 [20, 47] |  | 34 [26, 43] | 36 [29, 48] | 37 [27, 58] |  |
| Platelet value | Day 1 |  |  |  | <0.001 |  |  |  | <0.001 |  |  |  | <0.001 |  |  |  | <0.001 |
|  | Median [IQR] | 228 [194, 262] | 329 [292, 383] | 163 [126, 208] |  | 285 [234, 341] | 190 [150, 233] | 75 [48, 113] |  | 549 [474, 549] | 358 [301, 435] | 140 [74, 202] |  | 488 [403, 549] | 292 [234, 341] | 117 [69, 185] |  |
|  | Day 2 |  |  |  | <0.001 |  |  |  | <0.001 |  |  |  | <0.001 |  |  |  | <0.001 |
|  | Median [IQR] | 176 [147, 208] | 254 [216, 297] | 110 [76, 141] |  | 344 [289, 398] | 222 [178, 267] | 77 [47, 122] |  | 549 [487, 549] | 367 [297, 434] | 135 [77, 198] |  | 476 [399, 549] | 300 [241, 345] | 100 [64, 161] |  |
|  | Day 3 |  |  |  | <0.001 |  |  |  | <0.001 |  |  |  | <0.001 |  |  |  | <0.001 |
|  | Median [IQR] | 139 [111, 170] | 209 [155, 242] | 77 [53, 102] |  | 392 [338, 454] | 250 [201, 296] | 84 [48, 125] |  | 549 [477, 549] | 350 [290, 418] | 130 [66, 184] |  | 468 [414, 542] | 299 [246, 351] | 100 [54, 156] |  |
|  | Day 4 |  |  |  | <0.001 |  |  |  | <0.001 |  |  |  | <0.001 |  |  |  | <0.001 |
|  | Median [IQR] | 127 [96, 159] | 184 [137, 230] | 66 [48, 89] |  | 444 [382, 504] | 277 [224, 327] | 94 [51, 135] |  | 549 [468, 554] | 338 [278, 405] | 119 [55, 180] |  | 472 [411, 544] | 305 [252, 354] | 94 [46, 156] |  |
|  | Day 5 |  |  |  | <0.001 |  |  |  | <0.001 |  |  |  | <0.001 |  |  |  | <0.001 |
|  | Median [IQR] | 143 [111, 173] | 194 [146, 241] | 72 [53, 93] |  | 480 [417, 549] | 299 [245, 349] | 98 [58, 141] |  | 524 [449, 549] | 323 [264, 389] | 110 [49, 176] |  | 482 [424, 539] | 312 [259, 366] | 90 [42, 156] |  |
|  | Day 6 |  |  |  | <0.001 |  |  |  | <0.001 |  |  |  | <0.001 |  |  |  | <0.001 |
|  | Median [IQR] | 167 [134, 198] | 226 [172, 267] | 80 [56, 104] |  | 509 [446, 549] | 322 [272, 366] | 112 [69, 162] |  | 510 [439, 549] | 304 [241, 356] | 98 [43, 166] |  | 482 [417, 549] | 324 [265, 366] | 88 [47, 152] |  |
|  | Day 7 |  |  |  | <0.001 |  |  |  | <0.001 |  |  |  | <0.001 |  |  |  | <0.001 |
|  | Median [IQR] | 200 [167, 238] | 264 [210, 311] | 96 [64, 127] |  | 540 [473, 561] | 342 [293, 390] | 124 [75, 179] |  | 488 [410, 549] | 289 [232, 340] | 86 [34, 146] |  | 483 [420, 549] | 329 [270, 381] | 89 [47, 158] |  |
| Mean of overall median value | Platelet |  |  |  | <0.001 |  |  |  | <0.001 |  |  |  | <0.001 |  |  |  | <0.001 |
|  | Median [IQR] | 167 [127, 207] | 235 [177, 291] | 87 [60, 124] |  | 432 [345, 517] | 269 [210, 330] | 94 [54, 140] |  | 534 [457, 549] | 332 [270, 397] | 116 [54, 181] |  | 477 [414, 547] | 309 [255, 358] | 97 [52, 160] |  |

## Table S11. For lymphocyte

|  | | **Week 1** | | | | **Week 2** | | | | **Week 3** | | | | **Week 4** | | | |
| --- | --- | --- | --- | --- | --- | --- | --- | --- | --- | --- | --- | --- | --- | --- | --- | --- | --- |
| **Group** | **Variables** | **A**, N = 387 (22.4%) | **B**, N = 710 (41.1%) | **C**, N = 630 (36.5%) | **p-value** | **A**, N = 283 (22.3%) | **B**, N = 554 (43.7%) | **C**, N = 431 (34.0%) | **p-value** | **A**, N = 241 (27.3%) | **B**, N = 384 (43.5%) | **C**, N = 257 (29.1%) | **p-value** | **A**, N = 173 (26.1%) | **B**, N = 284 (42.9%) | **C**, N = 205 (31.0%) | **p-value** |
| Demographics | Mortality | 22 (5.7%) | 185 (26.1%) | 182 (28.9%) | <0.001 | 23 (8.1%) | 71 (12.8%) | 210 (48.7%) | <0.001 | 14 (5.8%) | 49 (12.8%) | 125 (48.6%) | <0.001 | 6 (3.5%) | 30 (10.6%) | 76 (37.1%) | <0.001 |
|  | Patient Age |  |  |  | <0.001 |  |  |  | <0.001 |  |  |  | <0.001 |  |  |  | 0.025 |
|  | Median [IQR] | 48 [38, 56] | 51 [41, 62] | 50 [41, 59] |  | 48 [39, 58] | 49 [40, 58] | 54 [44, 66] |  | 48 [40, 57] | 51 [41, 60] | 54 [43, 65] |  | 49 [42, 58] | 50 [41, 61] | 53 [44, 66] |  |
|  | Sex |  |  |  | 0.458 |  |  |  | 0.865 |  |  |  | 0.701 |  |  |  | 0.216 |
|  | Male | 309 (79.8%) | 586 (82.5%) | 507 (80.5%) |  | 225 (79.5%) | 440 (79.4%) | 348 (80.7%) |  | 194 (80.5%) | 303 (78.9%) | 199 (77.4%) |  | 140 (80.9%) | 227 (79.9%) | 152 (74.1%) |  |
|  | Female | 78 (20.2%) | 124 (17.5%) | 123 (19.5%) |  | 58 (20.5%) | 114 (20.6%) | 83 (19.3%) |  | 47 (19.5%) | 81 (21.1%) | 58 (22.6%) |  | 33 (19.1%) | 57 (20.1%) | 53 (25.9%) |  |
|  | TBSA |  |  |  | <0.001 |  |  |  | <0.001 |  |  |  | <0.001 |  |  |  | <0.001 |
|  | Median [IQR] | 22 [10, 32] | 32 [20, 51] | 34 [22, 60] |  | 25 [15, 37] | 33 [22, 47] | 48 [28, 67] |  | 31 [19, 42] | 37 [24, 51] | 46 [31, 64] |  | 35 [21, 47] | 38 [25, 51] | 41 [26, 61] |  |
|  | Inhalation | 132 (34.1%) | 319 (44.9%) | 313 (49.7%) | <0.001 | 97 (34.3%) | 258 (46.6%) | 220 (51.0%) | <0.001 | 110 (45.6%) | 172 (44.8%) | 115 (44.7%) | 0.977 | 76 (43.9%) | 133 (46.8%) | 100 (48.8%) | 0.647 |
|  | LOICU |  |  |  | <0.001 |  |  |  | <0.001 |  |  |  | 0.006 |  |  |  | <0.001 |
|  | Median [IQR] | 9 [5, 22] | 17 [8, 32] | 18 [8, 34] |  | 17 [11, 28] | 26 [14, 40] | 22 [13, 36] |  | 28 [20, 38] | 30 [23, 44] | 30 [21, 47] |  | 32 [27, 41] | 36 [28, 52] | 38 [28, 58] |  |
| Lymphocyte value | Day 1 |  |  |  | <0.001 |  |  |  | <0.001 |  |  |  | <0.001 |  |  |  | <0.001 |
|  | Median [IQR] | 1.40 [1.14, 1.90] | 0.80 [0.55, 1.03] | 1.99 [1.60, 2.20] |  | 1.66 [1.40, 2.06] | 0.90 [0.70, 1.10] | 0.52 [0.36, 0.70] |  | 1.43 [1.19, 1.69] | 0.90 [0.74, 1.09] | 0.50 [0.36, 0.66] |  | 1.48 [1.26, 1.79] | 0.97 [0.80, 1.12] | 0.60 [0.43, 0.77] |  |
|  | Day 2 |  |  |  | <0.001 |  |  |  | <0.001 |  |  |  | <0.001 |  |  |  | <0.001 |
|  | Median [IQR] | 1.58 [1.25, 1.97] | 0.93 [0.69, 1.22] | 1.40 [1.07, 1.80] |  | 1.71 [1.47, 2.08] | 1.01 [0.81, 1.24] | 0.59 [0.39, 0.75] |  | 1.43 [1.22, 1.70] | 0.91 [0.76, 1.09] | 0.53 [0.40, 0.68] |  | 1.47 [1.30, 1.78] | 0.98 [0.80, 1.13] | 0.60 [0.42, 0.76] |  |
|  | Day 3 |  |  |  | <0.001 |  |  |  | <0.001 |  |  |  | <0.001 |  |  |  | <0.001 |
|  | Median [IQR] | 1.40 [1.14, 1.69] | 0.80 [0.58, 1.07] | 1.00 [0.73, 1.37] |  | 1.67 [1.47, 2.05] | 1.05 [0.86, 1.27] | 0.58 [0.40, 0.76] |  | 1.44 [1.25, 1.70] | 0.92 [0.78, 1.10] | 0.50 [0.37, 0.66] |  | 1.47 [1.29, 1.81] | 0.98 [0.82, 1.14] | 0.60 [0.41, 0.75] |  |
|  | Day 4 |  |  |  | <0.001 |  |  |  | <0.001 |  |  |  | <0.001 |  |  |  | <0.001 |
|  | Median [IQR] | 1.22 [0.98, 1.54] | 0.62 [0.42, 0.87] | 0.76 [0.52, 1.00] |  | 1.66 [1.39, 1.97] | 1.06 [0.89, 1.29] | 0.57 [0.40, 0.75] |  | 1.44 [1.26, 1.73] | 0.91 [0.74, 1.09] | 0.50 [0.36, 0.66] |  | 1.50 [1.32, 1.78] | 1.00 [0.84, 1.18] | 0.60 [0.45, 0.75] |  |
|  | Day 5 |  |  |  | <0.001 |  |  |  | <0.001 |  |  |  | <0.001 |  |  |  | <0.001 |
|  | Median [IQR] | 1.18 [0.92, 1.46] | 0.56 [0.39, 0.80] | 0.64 [0.46, 0.90] |  | 1.63 [1.31, 2.02] | 1.06 [0.90, 1.27] | 0.55 [0.35, 0.70] |  | 1.42 [1.22, 1.70] | 0.92 [0.79, 1.10] | 0.51 [0.37, 0.70] |  | 1.53 [1.36, 1.89] | 1.04 [0.90, 1.21] | 0.60 [0.43, 0.75] |  |
|  | Day 6 |  |  |  | <0.001 |  |  |  | <0.001 |  |  |  | <0.001 |  |  |  | <0.001 |
|  | Median [IQR] | 1.30 [1.04, 1.56] | 0.58 [0.40, 0.79] | 0.65 [0.47, 0.90] |  | 1.58 [1.29, 1.91] | 1.04 [0.87, 1.24] | 0.54 [0.36, 0.70] |  | 1.44 [1.23, 1.77] | 0.96 [0.80, 1.18] | 0.54 [0.40, 0.70] |  | 1.56 [1.40, 1.94] | 1.07 [0.90, 1.23] | 0.59 [0.44, 0.75] |  |
|  | Day 7 |  |  |  | <0.001 |  |  |  | <0.001 |  |  |  | <0.001 |  |  |  | <0.001 |
|  | Median [IQR] | 1.50 [1.28, 1.77] | 0.65 [0.44, 0.90] | 0.72 [0.50, 0.98] |  | 1.53 [1.23, 1.90] | 1.03 [0.84, 1.23] | 0.50 [0.32, 0.68] |  | 1.47 [1.26, 1.81] | 0.96 [0.79, 1.12] | 0.51 [0.38, 0.70] |  | 1.60 [1.38, 2.00] | 1.10 [0.94, 1.28] | 0.61 [0.44, 0.78] |  |
| Mean of overall median value | Lymphocyte |  |  |  | <0.001 |  |  |  | <0.001 |  |  |  | <0.001 |  |  |  | <0.001 |
|  | Median [IQR] | 1.37 [1.09, 1.69] | 0.70 [0.47, 0.96] | 0.90 [0.60, 1.40] |  | 1.65 [1.37, 2.00] | 1.02 [0.83, 1.24] | 0.55 [0.36, 0.71] |  | 1.44 [1.23, 1.72] | 0.92 [0.77, 1.10] | 0.50 [0.37, 0.68] |  | 1.52 [1.32, 1.85] | 1.01 [0.86, 1.20] | 0.60 [0.43, 0.76] |  |

## Table S12. For lactate

|  | | **Week 1** | | | | **Week 2** | | | | **Week 3** | | | | **Week 4** | | | |
| --- | --- | --- | --- | --- | --- | --- | --- | --- | --- | --- | --- | --- | --- | --- | --- | --- | --- |
| **Group** | **Variables** | **A**, N = 432 (25.2%) | **B**, N = 618 (36.0%) | **C**, N = 666 (38.8%) | **p-value** | **A**, N = 650 (51.5%) | **B**, N = 475 (37.6%) | **C**, N = 138 (10.9%) | **p-value** | **A**, N = 414 (47.0%) | **B**, N = 339 (38.5%) | **C**, N = 128 (14.5%) | **p-value** | **A**, N = 333 (50.3%) | **B**, N = 239 (36.1%) | **C**, N = 90 (13.6%) | **p-value** |
| Demographics | Mortality | 14 (3.2%) | 83 (13.4%) | 291 (43.7%) | <0.001 | 95 (14.6%) | 121 (25.5%) | 87 (63.0%) | <0.001 | 43 (10.4%) | 72 (21.2%) | 72 (56.2%) | <0.001 | 20 (6.0%) | 44 (18.4%) | 48 (53.3%) | <0.001 |
|  | Patient Age |  |  |  | 0.130 |  |  |  | 0.603 |  |  |  | 0.006 |  |  |  | 0.411 |
|  | Median [IQR] | 49 [40, 58] | 51 [40, 61] | 51 [41, 59] |  | 51 [42, 61] | 50 [41, 59] | 51 [41, 61] |  | 50 [41, 62] | 52 [44, 61] | 48 [37, 58] |  | 51 [42, 62] | 52 [42, 61] | 50 [41, 59] |  |
|  | Sex |  |  |  | 0.275 |  |  |  | 0.898 |  |  |  | 0.771 |  |  |  | >0.999 |
|  | Male | 357 (82.6%) | 509 (82.4%) | 528 (79.3%) |  | 523 (80.5%) | 379 (79.8%) | 109 (79.0%) |  | 329 (79.5%) | 268 (79.1%) | 98 (76.6%) |  | 261 (78.4%) | 187 (78.2%) | 71 (78.9%) |  |
|  | Female | 75 (17.4%) | 109 (17.6%) | 138 (20.7%) |  | 127 (19.5%) | 96 (20.2%) | 29 (21.0%) |  | 85 (20.5%) | 71 (20.9%) | 30 (23.4%) |  | 72 (21.6%) | 52 (21.8%) | 19 (21.1%) |  |
|  | TBSA |  |  |  | <0.001 |  |  |  | <0.001 |  |  |  | <0.001 |  |  |  | <0.001 |
|  | Median [IQR] | 20 [10, 30] | 30 [20, 42] | 41 [25, 66] |  | 29 [20, 42] | 40 [26, 60] | 52 [33, 80] |  | 30 [20, 42] | 40 [30, 60] | 56 [37, 70] |  | 31 [20, 45] | 40 [30, 58] | 58 [40, 67] |  |
|  | Inhalation | 163 (37.7%) | 217 (35.1%) | 380 (57.1%) | <0.001 | 270 (41.5%) | 225 (47.4%) | 77 (55.8%) | 0.006 | 179 (43.2%) | 163 (48.1%) | 54 (42.2%) | 0.331 | 145 (43.5%) | 122 (51.0%) | 42 (46.7%) | 0.208 |
|  | LOICU |  |  |  | <0.001 |  |  |  | <0.001 |  |  |  | <0.001 |  |  |  | 0.009 |
|  | Median [IQR] | 9 [5, 20] | 19 [8, 32] | 19 [9, 36] |  | 22 [13, 35] | 26 [16, 39] | 13 [10, 25] |  | 29 [21, 40] | 34 [24, 48] | 23 [19, 35] |  | 35 [27, 47] | 38 [30, 54] | 31 [25, 47] |  |
| Lactate value | Day 1 |  |  |  | <0.001 |  |  |  | <0.001 |  |  |  | <0.001 |  |  |  | <0.001 |
|  | Median [IQR] | 1.50 [1.10, 1.90] | 3.00 [2.50, 3.50] | 5.70 [4.90, 5.70] |  | 1.19 [0.90, 1.50] | 2.30 [1.90, 2.72] | 3.70 [2.90, 4.50] |  | 1.20 [0.90, 1.50] | 2.20 [1.78, 2.64] | 3.30 [2.40, 4.22] |  | 1.21 [1.00, 1.50] | 2.30 [1.90, 2.69] | 3.60 [2.79, 4.40] |  |
|  | Day 2 |  |  |  | <0.001 |  |  |  | <0.001 |  |  |  | <0.001 |  |  |  | <0.001 |
|  | Median [IQR] | 1.40 [1.00, 1.90] | 2.80 [2.10, 3.50] | 4.40 [3.30, 5.70] |  | 1.20 [0.90, 1.50] | 2.35 [1.96, 2.80] | 3.80 [3.03, 4.57] |  | 1.20 [0.90, 1.50] | 2.27 [1.93, 2.70] | 3.60 [2.60, 4.53] |  | 1.20 [1.00, 1.50] | 2.30 [1.90, 2.70] | 3.70 [2.83, 4.53] |  |
|  | Day 3 |  |  |  | <0.001 |  |  |  | <0.001 |  |  |  | <0.001 |  |  |  | <0.001 |
|  | Median [IQR] | 1.30 [0.90, 1.70] | 2.10 [1.40, 2.77] | 3.00 [2.10, 4.00] |  | 1.20 [0.90, 1.50] | 2.40 [2.00, 2.80] | 4.10 [3.30, 5.70] |  | 1.20 [0.90, 1.50] | 2.27 [1.99, 2.70] | 3.67 [2.88, 5.00] |  | 1.20 [0.93, 1.50] | 2.30 [1.94, 2.70] | 3.84 [3.10, 5.70] |  |
|  | Day 4 |  |  |  | <0.001 |  |  |  | <0.001 |  |  |  | <0.001 |  |  |  | <0.001 |
|  | Median [IQR] | 1.20 [0.90, 1.70] | 1.80 [1.30, 2.40] | 2.40 [1.70, 3.40] |  | 1.20 [0.90, 1.50] | 2.35 [1.95, 2.80] | 4.08 [3.50, 5.68] |  | 1.20 [0.90, 1.50] | 2.20 [1.90, 2.67] | 3.87 [2.86, 5.69] |  | 1.18 [0.90, 1.44] | 2.30 [1.98, 2.70] | 4.20 [3.27, 5.70] |  |
|  | Day 5 |  |  |  | <0.001 |  |  |  | <0.001 |  |  |  | <0.001 |  |  |  | <0.001 |
|  | Median [IQR] | 1.12 [0.80, 1.50] | 1.60 [1.10, 2.29] | 2.10 [1.50, 3.00] |  | 1.20 [0.92, 1.50] | 2.34 [2.00, 2.80] | 4.17 [3.55, 5.70] |  | 1.20 [0.93, 1.50] | 2.23 [1.90, 2.66] | 3.92 [2.84, 5.70] |  | 1.15 [0.90, 1.46] | 2.30 [1.90, 2.70] | 4.23 [3.46, 5.69] |  |
|  | Day 6 |  |  |  | <0.001 |  |  |  | <0.001 |  |  |  | <0.001 |  |  |  | <0.001 |
|  | Median [IQR] | 1.10 [0.80, 1.50] | 1.55 [1.05, 2.23] | 2.10 [1.40, 3.16] |  | 1.17 [0.90, 1.47] | 2.30 [2.00, 2.70] | 4.10 [3.51, 5.64] |  | 1.20 [0.91, 1.50] | 2.20 [1.90, 2.76] | 3.82 [3.02, 5.68] |  | 1.16 [0.90, 1.47] | 2.30 [1.99, 2.70] | 4.14 [3.45, 5.66] |  |
|  | Day 7 |  |  |  | <0.001 |  |  |  | <0.001 |  |  |  | <0.001 |  |  |  | <0.001 |
|  | Median [IQR] | 1.12 [0.80, 1.52] | 1.60 [1.03, 2.30] | 2.20 [1.42, 3.30] |  | 1.17 [0.90, 1.50] | 2.30 [2.00, 2.80] | 4.30 [3.66, 5.68] |  | 1.20 [0.90, 1.48] | 2.21 [1.84, 2.69] | 4.33 [3.53, 5.70] |  | 1.14 [0.90, 1.45] | 2.30 [1.94, 2.70] | 4.30 [3.50, 5.67] |  |
| Mean of overall median value | Lactate |  |  |  | <0.001 |  |  |  | <0.001 |  |  |  | <0.001 |  |  |  | <0.001 |
|  | Median [IQR] | 1.28 [0.90, 1.70] | 2.03 [1.30, 2.80] | 2.90 [1.86, 4.70] |  | 1.20 [0.90, 1.50] | 2.32 [1.99, 2.80] | 4.08 [3.40, 5.68] |  | 1.20 [0.90, 1.50] | 2.21 [1.90, 2.70] | 3.80 [2.80, 5.69] |  | 1.20 [0.90, 1.50] | 2.30 [1.92, 2.70] | 3.90 [3.10, 5.67] |  |

## Table S13. For albumin

|  | | **Week 1** | | | | **Week 2** | | | | **Week 3** | | | | **Week 4** | | | |
| --- | --- | --- | --- | --- | --- | --- | --- | --- | --- | --- | --- | --- | --- | --- | --- | --- | --- |
| **Group** | **Variables** | **A**, N = 306 (17.7%) | **B**, N = 1025 (59.4%) | **C**, N = 394 (22.8%) | **p-value** | **A**, N = 172 (13.6%) | **B**, N = 571 (45.1%) | **C**, N = 524 (41.4%) | **p-value** | **A**, N = 134 (15.2%) | **B**, N = 429 (48.7%) | **C**, N = 318 (36.1%) | **p-value** | **A**, N = 111 (16.8%) | **B**, N = 345 (52.1%) | **C**, N = 206 (31.1%) | **p-value** |
| Demographics | Mortality | 4 (1.3%) | 166 (16.2%) | 218 (55.3%) | <0.001 | 3 (1.7%) | 54 (9.5%) | 246 (46.9%) | <0.001 | 2 (1.5%) | 46 (10.7%) | 139 (43.7%) | <0.001 | 1 (0.9%) | 26 (7.5%) | 85 (41.3%) | <0.001 |
|  | Patient Age |  |  |  | 0.004 |  |  |  | 0.043 |  |  |  | <0.001 |  |  |  | <0.001 |
|  | Median [IQR] | 48 [38, 56] | 50 [41, 60] | 51 [42, 61] |  | 48 [39, 59] | 51 [41, 60] | 52 [42, 62] |  | 45 [36, 55] | 52 [43, 63] | 51 [42, 61] |  | 45 [38, 56] | 52 [44, 63] | 53 [41, 62] |  |
|  | Sex |  |  |  | 0.016 |  |  |  | 0.004 |  |  |  | 0.783 |  |  |  | 0.511 |
|  | Male | 248 (81.0%) | 851 (83.0%) | 301 (76.4%) |  | 133 (77.3%) | 438 (76.7%) | 441 (84.2%) |  | 105 (78.4%) | 335 (78.1%) | 255 (80.2%) |  | 85 (76.6%) | 267 (77.4%) | 167 (81.1%) |  |
|  | Female | 58 (19.0%) | 174 (17.0%) | 93 (23.6%) |  | 39 (22.7%) | 133 (23.3%) | 83 (15.8%) |  | 29 (21.6%) | 94 (21.9%) | 63 (19.8%) |  | 26 (23.4%) | 78 (22.6%) | 39 (18.9%) |  |
|  | TBSA |  |  |  | <0.001 |  |  |  | <0.001 |  |  |  | <0.001 |  |  |  | <0.001 |
|  | Median [IQR] | 11 [6, 20] | 30 [21, 45] | 53 [32, 75] |  | 14 [9, 23] | 31 [22, 42] | 50 [33, 67] |  | 21 [10, 31] | 35 [23, 45] | 55 [37, 67] |  | 25 [12, 40] | 38 [25, 50] | 50 [32, 63] |  |
|  | Inhalation | 123 (40.2%) | 423 (41.3%) | 217 (55.1%) | <0.001 | 77 (44.8%) | 244 (42.7%) | 253 (48.3%) | 0.172 | 62 (46.3%) | 178 (41.5%) | 156 (49.1%) | 0.111 | 50 (45.0%) | 155 (44.9%) | 104 (50.5%) | 0.423 |
|  | LOICU |  |  |  | <0.001 |  |  |  | <0.001 |  |  |  | <0.001 |  |  |  | <0.001 |
|  | Median [IQR] | 6 [4, 10] | 18 [9, 33] | 20 [11, 35] |  | 13 [9, 24] | 23 [14, 36] | 24 [14, 41] |  | 23 [18, 34] | 31 [23, 43] | 32 [21, 50] |  | 29 [24, 36] | 37 [29, 49] | 38 [27, 58] |  |
| Albumin value | Day 1 |  |  |  | <0.001 |  |  |  | <0.001 |  |  |  | <0.001 |  |  |  | <0.001 |
|  | Median [IQR] | 3.50 [3.50, 3.50] | 3.50 [3.30, 3.50] | 2.30 [1.90, 2.60] |  | 3.07 [2.90, 3.27] | 2.70 [2.50, 2.80] | 2.40 [2.20, 2.50] |  | 3.00 [2.80, 3.20] | 2.60 [2.50, 2.80] | 2.40 [2.20, 2.60] |  | 3.00 [2.90, 3.20] | 2.60 [2.50, 2.80] | 2.33 [2.10, 2.50] |  |
|  | Day 2 |  |  |  | <0.001 |  |  |  | <0.001 |  |  |  | <0.001 |  |  |  | <0.001 |
|  | Median [IQR] | 3.34 [3.10, 3.50] | 2.50 [2.00, 2.90] | 1.90 [1.90, 2.30] |  | 3.08 [2.91, 3.30] | 2.60 [2.50, 2.80] | 2.40 [2.21, 2.60] |  | 3.00 [2.85, 3.20] | 2.60 [2.50, 2.80] | 2.40 [2.20, 2.60] |  | 3.10 [2.93, 3.20] | 2.60 [2.50, 2.80] | 2.40 [2.20, 2.60] |  |
|  | Day 3 |  |  |  | <0.001 |  |  |  | <0.001 |  |  |  | <0.001 |  |  |  | <0.001 |
|  | Median [IQR] | 3.10 [2.90, 3.40] | 2.40 [2.10, 2.60] | 2.10 [1.90, 2.40] |  | 3.09 [2.99, 3.20] | 2.69 [2.50, 2.80] | 2.40 [2.20, 2.60] |  | 3.00 [2.90, 3.20] | 2.65 [2.50, 2.80] | 2.40 [2.10, 2.60] |  | 3.10 [2.92, 3.20] | 2.70 [2.50, 2.90] | 2.40 [2.20, 2.60] |  |
|  | Day 4 |  |  |  | <0.001 |  |  |  | <0.001 |  |  |  | <0.001 |  |  |  | <0.001 |
|  | Median [IQR] | 3.10 [2.90, 3.40] | 2.50 [2.15, 2.70] | 2.20 [1.90, 2.50] |  | 3.04 [2.98, 3.22] | 2.70 [2.51, 2.81] | 2.40 [2.20, 2.60] |  | 3.00 [2.83, 3.20] | 2.60 [2.50, 2.80] | 2.40 [2.19, 2.60] |  | 3.05 [2.94, 3.20] | 2.70 [2.55, 2.82] | 2.40 [2.20, 2.60] |  |
|  | Day 5 |  |  |  | <0.001 |  |  |  | <0.001 |  |  |  | <0.001 |  |  |  | <0.001 |
|  | Median [IQR] | 3.12 [2.90, 3.40] | 2.50 [2.27, 2.73] | 2.30 [2.00, 2.60] |  | 3.10 [2.92, 3.30] | 2.70 [2.60, 2.82] | 2.40 [2.22, 2.60] |  | 3.00 [2.88, 3.19] | 2.60 [2.50, 2.80] | 2.38 [2.10, 2.50] |  | 3.10 [2.96, 3.25] | 2.70 [2.60, 2.90] | 2.40 [2.14, 2.60] |  |
|  | Day 6 |  |  |  | <0.001 |  |  |  | <0.001 |  |  |  | <0.001 |  |  |  | <0.001 |
|  | Median [IQR] | 3.15 [2.94, 3.44] | 2.60 [2.40, 2.80] | 2.40 [2.10, 2.60] |  | 3.10 [2.92, 3.30] | 2.70 [2.55, 2.82] | 2.42 [2.22, 2.60] |  | 3.00 [2.87, 3.21] | 2.70 [2.50, 2.81] | 2.35 [2.10, 2.60] |  | 3.11 [2.97, 3.30] | 2.70 [2.60, 2.90] | 2.40 [2.12, 2.60] |  |
|  | Day 7 |  |  |  | <0.001 |  |  |  | <0.001 |  |  |  | <0.001 |  |  |  | <0.001 |
|  | Median [IQR] | 3.18 [2.95, 3.45] | 2.60 [2.40, 2.80] | 2.40 [2.20, 2.60] |  | 3.10 [2.93, 3.30] | 2.73 [2.60, 2.90] | 2.40 [2.20, 2.53] |  | 3.00 [2.89, 3.21] | 2.70 [2.50, 2.90] | 2.30 [2.00, 2.50] |  | 3.13 [2.94, 3.30] | 2.75 [2.60, 2.90] | 2.37 [2.10, 2.58] |  |
| Mean of overall median value | Albumin |  |  |  | <0.001 |  |  |  | <0.001 |  |  |  | <0.001 |  |  |  | <0.001 |
|  | Median [IQR] | 3.25 [3.00, 3.50] | 2.60 [2.30, 2.90] | 2.30 [1.90, 2.50] |  | 3.10 [2.92, 3.28] | 2.70 [2.50, 2.80] | 2.40 [2.20, 2.60] |  | 3.00 [2.86, 3.20] | 2.67 [2.50, 2.80] | 2.40 [2.10, 2.60] |  | 3.10 [2.93, 3.23] | 2.70 [2.52, 2.85] | 2.40 [2.13, 2.60] |  |

Click [here](https://rpubs.com/dohernBurn/988304) to see the Sankey Diagram for Association Biomarkers, Clusters, Latent Class and Mortality.
a
